# Early transcriptional and cellular abnormalities in choroid plexus of a mouse model of Alzheimer’s disease

**DOI:** 10.1186/s13024-025-00853-w

**Published:** 2025-05-31

**Authors:** Zhong-Jiang Yan, Maosen Ye, Jiexi Li, Deng-Feng Zhang, Yong-Gang Yao

**Affiliations:** 1 State Key Laboratory of Genetic Evolution & Animal Models, Yunnan Key Laboratory of Animal Models and Human Disease Mechanisms, and KIZ-CUHK Joint Laboratory of Bioresources and Molecular Research in Common Diseases, Kunming Institute of Zoology, Chinese Academy of Sciences, Kunming, Yunnan 650204 China; 2 Kunming College of Life Science, University of Chinese Academy of Sciences, Kunming, Yunnan 650204 China; 3 National Research Facility for Phenotypic & Genetic Analysis of Model Animals (Primate Facility), National Resource Center for Non-Human Primates, Yunnan Engineering Center on Brain Disease Models, Kunming Institute of Zoology, Chinese Academy of Sciences, Kunming, Yunnan 650107 China

**Keywords:** Alzheimer’s disease, Choroid plexus, Single-cell transcriptome, Lipid accumulation, Neuroinflammation

## Abstract

**Background:**

Alzheimer’s disease (AD) is a progressive neurodegenerative disorder characterized by the accumulation of amyloid-β plaques, tau hyperphosphorylation, and neuroinflammation. The choroid plexus (ChP), serving as the blood-cerebrospinal fluid-brain barrier, plays essential roles in immune response to stress and brain homeostasis. However, the cellular and molecular contributions of the ChP to AD progression remain inadequately understood.

**Methods:**

To elucidate the molecular abnormalities during the early stages of AD, we acquired single-cell transcription profiling of ChP from APP/PS1 mice with early-stage of Aβ pathology and litter-mate controls. The transcriptional alterations that occurred in each cell type were identified by differentially expressed genes, cell–cell communications and pseudotemporal trajectory analysis. The findings were subsequently validated by a series of in situ and in vitro assays.

**Results:**

We constructed a comprehensive atlas of ChP at single-cell resolution and identified six major cell types and immune subclusters in male mice. The majority of dysregulated genes were found in the epithelial cells of APP/PS1 mice in comparison to wild-type (WT) mice, and most of these genes belonged to down-regulated module involved in mitochondrial respirasome assembly, cilium organization, and barrier integrity. The disruption of the epithelial barrier resulted in the downregulation of macrophage migration inhibitory factor (MIF) secretion in APP/PS1 mice, leading to macrophage activation and increased phagocytosis of Aβ. Concurrently, ligands (e.g., APOE) secreted by macrophages and other ChP cells facilitated the entry of lipids into ependymal cells, leading to lipid accumulation and the activation of microglia in the brain parenchyma in APP/PS1 mice compared to WT controls.

**Conclusions:**

Taken together, these data profiled early transcriptional and cellular abnormalities of ChP within an AD mouse model, providing novel insights of cerebral vasculature into the pathobiology of AD.

**Supplementary Information:**

The online version contains supplementary material available at 10.1186/s13024-025-00853-w.

## Background

Alzheimer disease (AD), the most prevalent cause of dementia, is biologically defined by the accumulation of amyloid-β (Aβ) plaques, tau aggregates, and neurodegeneration, leading to neuronal loss and synaptic dysfunction [1, 2]. These pathological features are predominantly identified at the late stages of AD, with a primary focus on the brain parenchyma, where neurons, microglia, and astrocytes are more affected. Despite extensive research, effective parenchyma-based therapeutic strategies for AD have yet to be developed, largely due to the complexity and prolonged duration of the disease [3–8]. Therefore, exploring the abnormalities that arise during the initial stages of AD, particularly those occurring outside the parenchyma, may provide valuable insights into AD progression.

Systematic environmental dyshomeostasis could precipitate cellular dysfunction within the parenchyma, which is intimately associated with cerebrospinal fluid (CSF). Biomarkers such as reduced levels of Aβ and increased levels of total tau (T-tau) or tau phosphorylated at threonine 181 (p-Tau181) in CSF are well-established diagnostic indicators of AD [9, 10]. Longitudinal studies have indicated that lower levels of Aβ_42_ in CSF can be detected as early as 18 years before the clinical manifestation of AD symptoms [11], alongside an elevated presence of AD-related proteins, such as apolipoprotein E (APOE), clusterin (CLU), Aβ precursor protein (APP), and complement component 7 (C7), all of which are implicated in Aβ clearance [12, 13], immunological surveillance, and protection [14]. These observations suggest an early disruption of CSF homeostasis, as well as perturbations in the related vasculature and lymphatic drainage systems in AD [15–18]. The choroid plexus (ChP), located along the ventricles of the brain, is the primary site of CSF production [19, 20] and functions as the blood-CSF barrier. Comprising blood vessels and specialized epithelial cells equipped with cilia, channels, and transporters, the ChP plays a crucial role in peripheral and nervous system maintenance [21]. Recent clinical studies have identified a ChP-dysfunction-associated AD subtype, characterized by increased expression of ChP-related protein involved in inflammation, lipid metabolism, and amyloid metabolism [22]. Moreover, magnetic resonance imaging (MRI) has revealed an increase in ChP volume in AD patients [23–25], suggesting a link between ChP dysfunction and AD pathology. Bulk transcriptional analyses of the ChP in AD have highlighted pathological changes in immune response [26] and CSF homeostasis [27], which may arise secondary to Aβ-induced toxicity [28] and compromised blood-CSF barrier integrity [29]. The ChP also mediates the entry of Aβ-activated peripheral monocytes into the brain parenchyma [30, 31]. Importantly, several AD-related genes identified through genome-wide association studies [32] have been shown to play a regulatory role in ChP function. For instance, *APOE* [33] and *TREM2* [34] are known to regulate inflammation and leukocyte infiltrations in the ChP.

The initial transcriptional and cellular atlas of the ChP has revealed that it is composed of epithelial, endothelial, mesenchymal, and immune cells [35]. The ChP shares transcriptional signatures with microglia in the brain parenchyma [36, 37], immune cells in the meninges [38], and cell types associated with the blood–brain barrier (BBB) [39, 40]. However, subsequent studies identified extensive ChP contamination in the sampling and profiling of human and rodent brain tissues in earlier datasets [41, 42], leading to potential misinterpretation of physiological and pathological phenomena, such as immune activation in AD [43–45]. As AD is a progressive neurodegenerative disease characterized by neuropathological abnormalities in biomarkers, particularly Aβ accumulation [46, 47], which can be detected up to two decades before clinical diagnosis, it is plausible that early Aβ exposure may impair ChP function [28, 29], thereby promoting AD-relevant pathology. Thus, investigating the subtle impacts on the ChP at single-cell resolution could provide valuable insights into the role of this complex barrier in AD development.

In this study, we generated gene expression profiles of 31,910 single cells isolated from the ChP of eight 4-month-old male APP/PS1 mice exhibiting early Aβ pathology together with that of eight wild-type (WT) littermates, enabling the characterization of early transcriptional changes in the ChP associated with AD. We found significant structural and functional impairments of ChP epithelial cells, which subsequently led to macrophage dysfunction. Specifically, ligands secreted by M2 macrophages in the ChP were found to interact with receptors in ependymal cells, regulating lipid endocytosis and uptake from the CSF—processes that may contribute to Aβ plaque formation and neuroinflammation in the parenchyma. These findings offer novel insights into the role of the ChP in the early stage of AD pathogenesis.

## Methods

### Animals

All animals were housed in a temperature-controlled environment under a 12-h light:12-h dark cycle and provided with food and water ad libitum. The APP/PS1 mice were originally obtained from the Jackson Laboratory (stock no.#34829-JAX, Bar Harbor, ME, USA). Before sampling for single-cell RNA sequencing (scRNA-seq) and validation assays, APP/PS1 and C57BL/6 mice were genotyped using standard polymerase chain reaction (PCR), with DNA extracted from tail biopsies using basic buffer under 95 ℃ for 1 h. Primers sequences were provided by the Jackson laboratory (protocol ID 33897). We mainly used male APP/PS1 mice and male WT mice for scRNA-seq (*n* = 8 per group) and experimental validations (up to *n* = 8 per group). A group of female APP/PS1 mice (*n* = 3) and female WT (*n* = 3) mice were also used for scRNA-seq. All mice used in this study were 4-month-old except for the cohort (8-month-old) used for immunostaining for Aβ-plaques. The study received ethical and scientific approval from the Kunming Institute of Zoology and strictly adhered to the animal care regulations of the Institutional Animal Care and Use Committee of Kunming Institute of Zoology.

### Isolation and dissociation of ChP

The mice were sacrificed by decapitation following anesthesia administered via intraperitoneal injection of tiletamine-zolazepam (20 μg/g) (Zoletil®50, Virbac Group) and xylazine (10 μg/g) (Xylazine hydrochloride injection, Jilin Huamu Animal Health Product Co., Ltd.). The brains were rapidly dissected and immediately placed into pre-chilled Dulbecco’s Modified Eagle Medium (DMEM) without phenol red (G4525, Servicebio). Because of the limited volume of ChP in a single mouse, ChP from the lateral ventricle and the fourth ventricle from three to five mice were carefully isolated and pooled into a 1.5 ml tube containing cold Singleron sCelLive™ tissue preservation solution on ice. Two biological replicates were obtained for male APP/PS1 and WT mice (batch 1, *n* = 5 per group; batch 2, *n* = 3 per group). The tissues were minced into small pieces with fine scissors inside the tube. Each sample was centrifuged at 300 g for 5 min at 4 ℃ and washed with Hank’s balanced salt solution (HBSS) (G4205, Servicebio), followed by digestion with Singleron sCelLive™ Tissue Dissociation Solution using the Singleron PythoN™ Tissue Dissociation System at 37 °C for 30 min. The cell suspension was collected and filtered through a 40 μm strainer. Red blood cells were removed by incubating the filtered mixture with GEXSCOPE® red blood cell lysis buffer (RCLB, Singleron) (volume of filtered mixture: volume of RCLB = 1:2) for 8 min at room temperature. Cells were centrifuged at 300 g for 5 min at 4 ℃, then gently resuspended in 200 μL of cold HBSS after removing the supernatant. Finally, cell viability and clumping were evaluated microscopically by trypan blue staining.

### Library construction and sequencing

The scRNA-seq library was constructed following previously reported procedures [48]. Briefly, 100 μL of suspended solution (2 × 10^5^ cells/mL) was loaded onto a microwell chip using the Singleron Matrix® Single Cell Processing System (Singleron Biotechnologies). The cDNA was subsequently obtained from the collected barcoding beads in the chip, followed by PCR amplification. The amplified cDNA was then fragmented and ligated with sequencing adapters, sequenced on the Illumina NovaSeq 6000 platform with 150 bp paired end reads.

### Raw data processing, quality control, and cell type identification

The raw scRNA-seq reads were processed to generate gene expression matrices using the *CeleScope* (https://github.com/singleron-RD/CeleScope) v1.2.1 pipeline. Initially, low-quality reads, poly-A tails, and adapter sequences were removed with *Cutadapt* v3.7 (https://github.com/marcelm/cutadapt/). Cell barcodes and unique molecular identifiers (UMI) were extracted to distinguish individual cells and transcripts. Subsequently, the reads were mapped to the reference genome GRCm39 (Ensembl v104 annotation) with STAR v2.6.1b [49]. UMI counts for each cell were obtained with the *featureCounts* function in the subread package v2.0.1 [50] and used to generate expression matrix files for subsequent analysis.

To obtain a preliminary assessment of data quality, cell types were identified before performing any data quality control using the R package Seurat v4.2.1 [51]. In brief, the raw UMI matrix was normalized using the *NormalizeData* function. Cell cycle phases were evaluated using the *CellCycleScoring* function, and data were scaled using the *ScaleData* function with the following variables regressed out: percent of mitochondrial gene expressed (percent.mito), number of genes expressed in a cell (nCount_RNA), number of transcripts expressed in a cell (nFeature_RNA), and cell cycle score (S.Score and G2M.Score). After data scaling, principal component analysis (PCA) was performed using the *RunPCA* function on the first 3 000 variable genes identified by the *FindVariableFeatures* function with the *vst* algorithm. Since we conducted two rounds of scRNA-seq for the ChP samples from male mice, potential batch effect was controlled using the anchor-based approach provided by Seurat [51]. The *RunUMAP* function was used to perform Uniform Manifold Approximation and Projection (UMAP) based on the top 20 principal components (PCs), with the *min.dist* parameter set to 0.1. After dimensionality reduction, the *FindNeighbors* function was used to construct a shared nearest neighbor graph by calculating neighborhood overlap between each cell based on the first two dimensions of UMAP. Cell clusters (original clusters identified by Seurat) were then identified using the *FindCluster* function (*methods* = graph, *resolution* = 0.05, *algorithm* = 4). Cluster-specific markers were identified using the *FindAllMarkers* function with default parameters, and cell types were identified based on canonical markers.

Following cell type identification, epithelial cells exhibited a high percentage of mitochondrial gene expression although no cell death signatures were observed. This indicated that a single cut-off for mitochondrial gene percentage or gene count was inadequate for this dataset. To address this problem, a cluster-specific manual quality control strategy was employed. Notably, each Seurat cluster identified in the previous step was extracted and subclustered using the same pipeline. Subclusters with a high proportion of mitochondrial gene expression and low gene counts were removed, as these were likely to represent potential dead cells. After manually removing these dead cells, all Seurat clusters were combined, and potential doublets were identified and removed.

Doublet removal was performed using the R package *DoubletFinder* v2.0 [52] with default parameters, with 15% set as the expected proportion of doublet cells. Doublets were identified by the dual expression of marker genes from different subclusters within a cluster (e.g., immunological genes and epithelial or endothelial genes) and were removed before further analysis and visualization. After doublet removal, data normalization, data scaling, dimensionality reduction, cell cluster identification, and cluster-specific marker identification were carried out using the aforementioned pipeline. Clusters were first categorized into broad cell types (e.g., immune cells, epithelial cells). The immune cells were then extracted and subclustered into more detailed categories based on immune cell markers.

### Differentially expressed genes (DEG) and enrichment analysis

To identify DEGs between APP/PS1 mice and controls, the Seurat *FindMarkers* function was used with the MAST method [53], applying the following parameters: *min.pct* = *0.01*, *logfc.threshold* = *0.01*, *latent.vars* = *"Batch"*, *assay* = *“RNA”*. DEGs were defined if *p_val_adj* < 0.05, and *|avg_log2 FC|*> 0.1. Gene Ontology (GO) biological process enrichment was performed using Metascape [54] and visualized by https://www.bioinformatics.com.cn.

### Cell–cell communication and pseudotime trajectory analyses

Cell–cell communications were identified using the R package *CellChat* [55]. To investigate potential differences in cellular communication among groups, scRNA-seq data were separated into APP/PS1 and WT control groups using the *SplitObject* function in Seurat, then analyzed according to the CellChat workflow [55]. To ensure the robustness of our analysis, only cell types or subtypes with a minimum of 100 cells were retained for cell–cell communication analysis. *CellChatDB* was modified to ensure that both ligands and receptors accurately represented their associated communications. Results from CellChat analysis of the two groups were merged using the *mergeCellChat* function, and differences between groups were visualized according to the standard CellChat processes, including interaction numbers, interaction strength, and signaling pathways between cell types. To identify communications significantly altered between APP/PS1 and WT mice, the *netMappingDEG* function was used. Receptor-ligand pairs that met the following criteria were designated as altered cell communications: |*ligand.logFC*|> 0.05, |*receptor.logFC*|> 0.05, *ligand.pvalues* < 0.05, and *receptor.pvalues* < 0.05. Adobe Illustrator 2023 was used to improve the contrast of visualizations.

Macrophage trajectory was constructed using the slingshot R package (https://github.com/kstreet13/slingshot). First, the Seurat object containing macrophages was converted to a SingleCellExperiment (sce) object using the *as.SingleCellExperiment* from Seurat [51]. The *slingshot* function was then applied to identify cell trajectories, with the parameters “*clusterLabels* = *'immune.celltype', reducedDim* = *'tree', dist.method* = *"slingshot", smoother* = *"smooth.spline", start.clus* = *"M2.mac", end.clus* = *"M1.mac"*”. Following trajectory construction, the gam function from mgcv R package (https://cran.r-project.org/web/packages/mgcv/index.html) was used to identify genes that were altered along the cell trajectory, with genes having a *q_value* < 0.05 designated as significant. M2 trajectory gene set was defined as the first cluster in the expression heatmap, while M2 gene set expression value was calculated by aggregating the expression level of every gene from the gene set for each cell.

### Immunostaining

As the original ChP tissues were fully utilized for scRNA-seq, we used independent samples from a separate cohort of mice for subsequent immunostaining. We used the left cerebral hemisphere for immunofluorescence staining, while the right hemisphere of the same animal was dissected into specific brain regions for protein isolation and lipoprotein particles measurement. All sections used for immunofluorescence staining were paraffin-embedded. Briefly, dissected tissues were immediately placed in 4% paraformaldehyde (PFA) diluted in phosphate-buffered saline (PBS) for 24 h, then fixed for another 24 h in fresh 4% PFA at room temperature. The tissues were subsequently dehydrated with gradient alcohol and cleared with alcohol-xylene for 10 min, xylene alone for 10 min, then infiltrated with melted paraffin at 65 ℃ three times and cooled on a pre-cold platform. All tissues were sectioned at a thickness of 4 μm for immunostaining. For immunostaining, the sections were twice subjected to deparaffinization buffer (G1128, Servicebio) and rehydrated with gradient alcohol. Antigen retrieval was performed using citrate antigen retrieval solution (pH 6.0) (MVS-0101, MXB biotechnologies) in a microwave for all antibodies. Briefly, after antigen repair and blocking in 5% bovine serum albumin (BSA) containing 5% donkey serum (SL050, Solarbio) or 5% goat serum, sections were incubated with primary antibodies for 12 h at 4 ℃, and secondary antibodies for 1.5 h at room temperature. All primary and secondary antibodies were provided in Supplementary Table S1. Finally, all slides were stained with 1 μg/mL 4’,6-diamidino-2-phenylindole (DAPI) (MB3204, Meilunbio) for 20 min to visualize cell nuclei and were sealed with anti-fluorescence quenching sealing agent (Fluoromount-G#0100-1, SouthernBiotech).

We also optimized a novel fluorescent multiplex immunohistochemical (mIHC) approach to identify cell types of ChP by integrating IHC with tyramide signal amplification (TSA) and a standard IHC protocol to co-stain OTX2, PECAM1, and ACTA2. Briefly, after antigen retrieval, endogenous peroxidase was blocked by incubating the sections with 3% H_2_O_2_ for 25 min away from light, followed by washing three times with PBS containing 1‰ Tween-20 (PBST) (9005-64-5, Sangon Biotech) for 5 min each. The sections were then blocked using 5% BSA (C16766338, Macklin) containing 5% goat serum (SL038, Solarbio), followed by incubation with rabbit anti-OTX2 for 12 h at 4℃. After washing three times with PBST, the sections were incubated with diluted horseradish peroxidase (HRP)-conjugated anti-rabbit secondary antibody for 1.5 h at room temperature. The slides were then placed in PBST and washed three times with PBST (5 min each time), followed by incubation with diluted iF488-TSA (G1231, Servicebio, 1:500) for 10 min at room temperature. After three additional PBST washes, unbound antibodies were removed by microwave treatment in citrate antigen retrieval solution (pH 6.0), which also performed antigen retrieval for subsequent staining. The sections were then incubated with the primary antibody rabbit anti-ACTA2 for 12 h at 4℃. After washing three times with PBST, the sections were incubated by incubation HRP-conjugated goat anti-rabbit secondary antibody for 1.5 h at room temperature, followed by incubation with iF647-TSA (G1232, Servicebio, 1:500) for 10 min at room temperature. After removal of the unbound antibodies, the sections were incubated with the primary antibody rabbit anti-CD31 for 12 h at 4 ℃, followed by incubation of rhodamine red-conjugated donkey anti-rabbit secondary antibody for 1.5 h at room temperature. All slides were stained with 1 μg/mL DAPI followed by sealing with anti-fluorescence quenching sealing agent as standard staining procedure. Imaging acquisition was conducted using TissueFAXS Spectra Systems integrated with StrataQuest quantitative analysis platform (TissueGnostics GmbH, Austria), allowing simultaneous multichannel fluorescence detection and automated image quantification.

### Mouse bone marrow-derived macrophages preparation, treatment, and detection

The Aβ monomers, fibrils, and oligomers were prepared following reported protocol with modifications [56]. Synthetic Aβ_42_ peptides (04010043259, China Peptides) were dissolved in hexafluoroisopropanol (HFIP, H107503, Aladdin) to 1 mM, incubated for 30 min at 25 °C, and vacuum-dried using a centrifugal concentrator (Concentrator plus, Eppendorf) to form transparent films. The films were reconstituted in dimethyl sulfoxide (DMSO, ST038, Beyotime) with pulsed sonication. To generate distinct aggregation states, monomers were prepared by diluting films in ice-cold ultrapure water (1 mM, stored at −80 °C); oligomers were resuspended in serum-free DMEM and incubated at 4 °C for 24 h; fibrils were formed in 10 mM HCl (pH 2.0) at 37 °C for 24 h.

Bone marrow-derived macrophages (BMDMs) were generated from 4-month-old male WT mice (*n* = 3) following our established protocol [57]. In brief, under sterile conditions, bone marrow cells from tibiae and femora were cultured in RPMI-1640 medium (11875093, Gibco) supplemented with 10% fetal bovine serum (10099–141, Gibco), 1% penicillin/streptomycin (15140122, Gibco), and 30% L929 culture supernatant (pre-treated with 50 μM β-mercaptoethanol, M274256, Sigma-Aldrich) conditioned medium for 7 days, with medium replacement on day 3 and day 5, respectively. Differentiated BMDMs were seeded in 6-well plates with 5% L929-conditioned medium. For Aβ stimulation experiments, cells were treated 12 h post-seeding with Aβ species (monomers/oligomers/fibrils; 0.2–20 μM) for 48 h. To investigate the role of macrophage migration inhibitory factor (MIF) in Aβ turnover and inflammation, BMDMs were pretreated for 6 h with either 500 ng/mL lipopolysaccharide (LPS, #L2630, Sigma) (washed after treatment of 6 h with PBS) and 5 μM Aβ aggregates, followed by 48 h exposure to medium containing 5% L929 culture supernatant which was pretreated with 100 μM 4-iodo-6-phenylpyrimidine(4-IPP, 41270966, MedChemExpress) for 48 h at 37℃. Culture supernatants were collected for cytokine profiling and Aβ characterization. Tumor necrosis factor-α (TNF-α) and interleukin-1β (IL-1β) levels were quantified using enzyme linked immunosorbent assay (ELISA) kits (TNF-α, EELM3063; IL-1β, EELM0037, Elabscience) according to manufacturer protocols. For Aβ immunoblotting, samples were heat-denatured (80 °C, 5 min), separated on 15% Tris–glycine SDS-PAGE gels, and electrotransferred to 0.22 μm polyvinylidene fluoride membranes at 200 mA for 40 min. Membranes were blocked with 5% non-fat milk and probed with 4G8 anti-Aβ monoclonal antibody (1:1,000 dilution, 800701, Biolegend) overnight at 4 °C, followed by horseradish peroxidase-conjugated secondary antibody incubation and chemiluminescent detection.

### Permeability measurement by intracerebroventricular injection of dextran

Four-month-old male APP/PS1 and WT mice (*n* = 5 for each group) were anesthetized and placed on a stereotaxic apparatus (68802, RWD Life Science), with 5 mg/mL dextran (D1818, ThermoFisher) subsequently injected into the right lateral ventricle at 500 nL/min for a total of 4.5 μL/mouse (coordinates from bregma: *AP* = − 0.58 mm, *ML* = + 1.0 mm, *DV* = − 2.0 mm). After injection for 30 min, blood samples were collected with EDTA anticoagulant tubes and placed at 4 ℃, then centrifuged at 3 000 g at 4 ℃ for 15 min to acquire plasma. The level of dextran in plasma was measured using a Synergy H1 Microplate Reader (BioTek, Agilent).

### Insoluble protein preparation and lipoprotein particle measurement

The insoluble protein fraction was determined following previously reported protocol [58]. All buffers used contained protease inhibitor cocktail. Samples from the cortex of 4-month-old male APP/PS1 and WT mice (*n* = 5 for each group) were placed into ultracentrifuge tubes (342303, Berkman) for centrifugation with an ultracentrifuge (Beckman Coulter, Optima™ Max-XP). Briefly, cortical tissue was homogenized in ice-cold Tris-Buffered Saline (TBS) and centrifuged at 175 000 g for 30 min at 4 ℃. The supernatant was collected as the TBS-soluble fraction, and the remaining pellets were washed with cold PBS. The washed pellets were then resuspended in TBS containing 1% Triton X-100 (TBST), gently mixed, and centrifuged again 175 000 g for 30 min at 4 ℃. The supernatant was collected as the TBST-soluble fraction, and the remaining pellets were washed with cold PBST. The pellets were then gently resuspended by 5 mol/L GuHCl, sonicated for 1 min, incubated for 12 h on ice, and centrifuged at 25 000 g for 30 min at 4 ℃. The resulting supernatant was collected as the GuHCl-insoluble fraction. The GuHCl-insoluble fractions were used for the measurement of high-density lipoprotein (HDL) (EM1113, FineTest), low-density lipoprotein (LDL) (EM1184, FineTest), and very low-density lipoprotein (VLDL) (EM1863, FineTest) using ELISA.

### Statistical analysis

Statistical analyses for validation experiments were performed using GraphPad Prism v9.0. Differences in cell-type distribution across groups were evaluated using chi-square tests. Two-tailed Student’s *t-*test with 95% confidence intervals (unpaired and parametric tests assuming Gaussian distribution) was used to compare differences between two groups, while two-way analysis of variance (ANOVA) was used to analyze differences in mean intensity away from the apical membrane among groups. Data are presented as mean ± standard deviation (SD) or mean ± standard error of the mean (SEM) as indicated in the figure legends. A *P-*value < 0.05 was considered statistically significant.

## Results

### Cellular and transcriptional characteristics of ChP in APP/PS1 mice

**Fig. 1:**
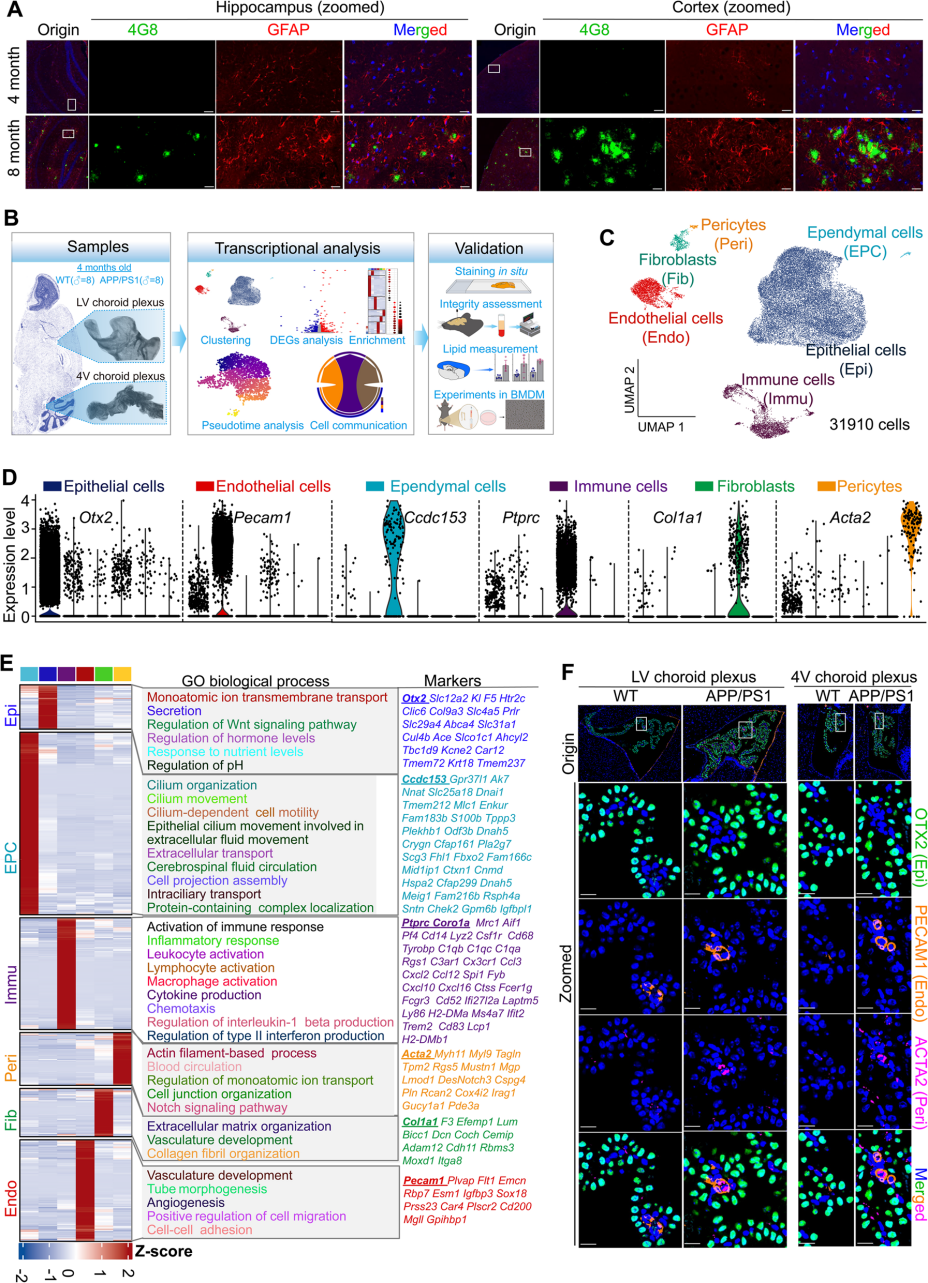
Single-cell transcriptional atlas of the ChP in APP/PS1 mice exhibiting early amyloid pathology. **A** Co-staining of Aβ with 4G8 (green) and astrocytes with GFAP (red) in the hippocampus and cortex of 4-month-old (*n* = 5 per group) and 8-month-old (*n* = 5 per group) APP/PS1 and WT mice, with nuclei stained by DAPI (blue). **B** Workflow for scRNA-seq of the ChP. Two pooled samples of ChP from the APP/PS1 (*n* = 8) and WT (*n* = 8) mice groups were sequenced. **C** UMAP of cell types identified in ChP of male APP/PS1 and WT mice. **D** Expression of conventional cell markers in broad cell types. The color label of each column corresponds to each cell type. **E** Cell markers, clustering, and GO biological processes, with underlined genes representing conventional or reported cell markers. The colors at the top of the clustering and the marker gene sets on the far right correspond to the cell type colors on the left, while the GO enrichment colors in the middle are purely for visual aesthetics and carry no specific meaning. LV, lateral ventricle; 4V, fourth ventricle. **F** Validation of typical cell type markers by in situ multiplex immunohistochemistry based on TSA method, with nuclei stained by DAPI (blue). Histological validations in (**F**) were performed on independent age-matched male cohorts (*n* = 8 per group) processed separately from those used for scRNA-seq, Scale bar, 20 μm. Origin in (**A** and **F**), original

Re-analysis of bulk transcriptomic datasets from multiple human brain regions [59] revealed that AD-associated genes reported in previous studies [32, 60] were more abundantly expressed in the ChP compared to other encephalic regions, including the cortex and hippocampus (Supplementary Fig. S1A). This observation suggested that the ChP may play a pivotal role in the pathological processes of AD. Given that amyloid plaques of APP/PS1 mice began to emerge in cortical tissues at 4-month-old (Fig. 1A), the ChP tissues were isolated from APP/PS1 and WT mice at this age. To ensure sufficient cells for scRNA-seq, ChP tissues from eight male mice in each group (male APP/PS1 group and male WT group) were pooled, with two biological replicates per group. After removal of poor-quality cells and doublets (Supplementary Fig. S1B), we observed minimal batch effects between the two replicates (Supplementary Figs. S1C-1D). We then pooled replicates in each group to increase the number of cells to achieve better statistical power. In total, 16, 650 and 15, 260 single-cell transcriptomes were obtained from male APP/PS1 mice and male WT mice, respectively (Supplementary Figs. S1E). The transcriptional features and alterations in the ChP between male APP/PS1 mice and controls were subsequently analyzed and validated (Fig. 1B).

Unsupervised clustering identified six broad cell types within the ChP, including epithelial, endothelial, and immune cells, each exhibiting distinct transcriptional profiles and functional roles (Fig. 1C-E and Supplementary Table S2). The identities of epithelial cells, endothelial cells, and pericytes were further validated through in situ co-staining of established cell type markers OTX2, PECAM1, and ACTA2 (Fig. 1F).

**Fig. 2:**
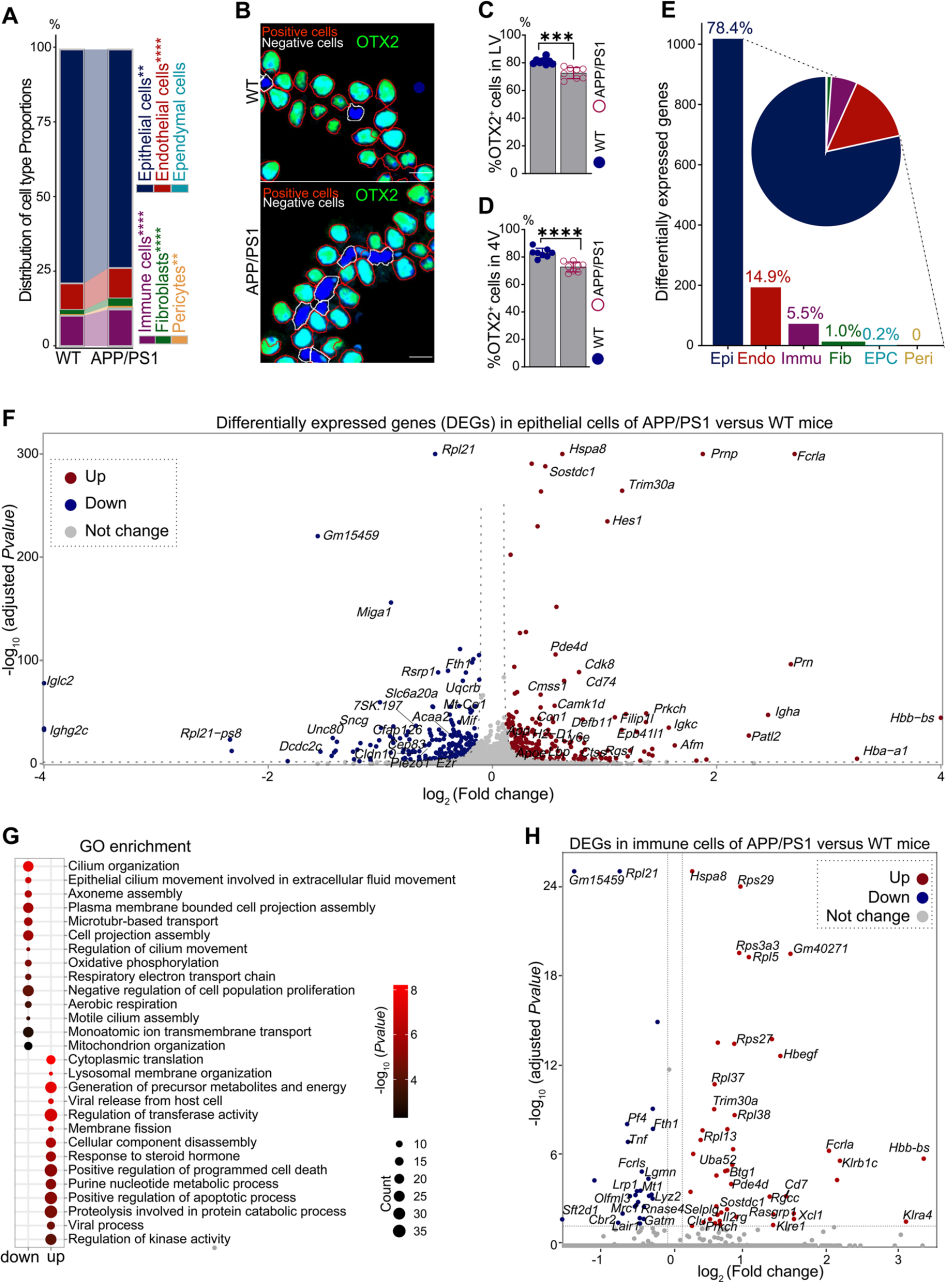
Cellular and transcriptional alterations in ChP of APP/PS1 mice. **A** Changes in cell ratio of each cell type in male APP/PS1 mice compared to wild-type (WT) mice. **B** Automatically and mechanically recognized epithelial cells (red box, OTX2-positive cells) and other cells (white box, OTX2-negative cells) based on staining signal of OTX2 (green fluorescence, specifically labeling epithelial cells) and DAPI (blue color, labeling all cell nuclei). Scale bar, 10 μm. **C-D** OTX2-positive cells (epithelial cells) were measured in the ChP of the lateral (LV, **C**) and the fourth ventricles (4V,** D**). Histological validations in (**B-D**) were performed on independent age-matched male cohorts (*n* = 8 per group) processed separately from those used for scRNA-seq. **E** Number and proportion of DEGs in each cell type. **F** Volcano plot showing distribution of epithelial DEGs. **G** Pathways enriched in up-regulated and down-regulated genes in epithelial cells. **H** Volcano plot showing distribution of DEGs in immune cells. Statistical analyses in (**A**) were performed using Chi-square test; Statistical analyses in (**C-D**) were performed using student’s t-test, and data were presented as mean ± SD; **, *p* < 0.01***; *p* < 0.001; ****, *p* < 0.0001

Leveraging the single-cell transcriptional atlas, we analyzed cell ratio changes and cell-type specific DEGs to uncover pathological changes in the ChP of male APP/PS1 mice. Epithelial cells constituted the majority of cells captured by single-cell sequencing, comprising up to 77.6% of the ChP population. Notably, a significant reduction in the ratio of epithelial cells was observed in male APP/PS1 mice compared to male WT mice (Fig. 2A). This decrease in epithelial cells was further corroborated by immunostaining and measurement of OTX2-positive cells in both the lateral and fourth ventricles (Fig. 2B-D).

In addition to cellular ratio changes, substantial transcriptomic dysregulation was observed in the epithelial cells. Among the 1,298 DEGs identified in all cell types, 1,018 (78.4%) were altered in epithelial cells, demonstrating the predominant role of epithelium in ChP dysregulation (Fig. 2E). The up-regulated DEGs were primarily associated with stress and inflammatory responses (e.g., *Hspa8*, *Fcrla*, MHC II complex, and *Trim30a*), whereas down-regulated genes were primarily involved in aerobic respiration (e.g., mitochondrial respirasome assembly including *Uqcrb*, *Mt-Co1* and *Mt-Co12*; mitochondrial lipid metabolism, *Acaa2*; and mitochondrial fusion, *Miga1*), cilium organization (e.g., *Cfap126* and *Cep83*), secretion and transport functions (e.g., *Slc6a20a* and *Fth1*), mechanosensation (e.g., *Piezo1*), interface integrity (e.g., *Ezr* and *Cldn10* for polarity or cell junction formation) and immune inhibition regulator (e.g., *Mif)* (Fig. 2F-G and Supplementary Table S3). In immune cells from the ChP, up-regulated genes (e.g., *Hspa8*, *Hbb-bs*, *Fcrla, Xcl1, Klrb1c, Klre1* and *Cd7*) were associated with inflammatory responses and immune activation, while down-regulated genes (e.g., *Pf4, Cbr2* and *Mrc1*) were primarily involved in the inhibition of inflammation (Fig. 2H). Consistent with patterns observed in immune and epithelial cells, endothelial cells from male APP/PS1 mice exhibited significant upregulation of MHC class II complexes (e.g., *H2-Eb1*, *H2Aa* and *H2-Ab1*) and immune-activating factors (e.g., *Fcrla, Pde4d*, *Cd74*, and *Apoe*), along with marked downregulation of basement membrane components (*Col4a1* and *Col4a2*) compared to male WT mice (Supplementary Fig. S3). These coordinated transcriptional shifts suggested compromised endothelial structural integrity coupled with enhanced immunoregulatory activation. Collectively, these transcriptomic alterations suggested that the ChP in male APP/PS1 mice exhibits impaired energy supply, disrupted cilium assembly, compromised secretion and barrier integrity, and altered stress responses, indicating an impairment of these signaling pathways at the early stage of amyloid pathology.

We also analyzed female APP/PS1 and WT mice, as sex difference in AD pathogenesis has been well recognized [61]. Different from male mice, scRNA-seq of ChP from female APP/PS1 (*n* = 7872 cells) and WT (*n* = 5681 cells) mice showed a higher ratio of epithelial cells in female mice than in male mice, and the epithelial cell ratio increased in the ChP tissues of female APP/PS1 mice compared to WT mice of same gender (Supplementary Figs. S3A-S3C). The majority of DEGs between female APP/PS1 mice and female WT mice were enriched in epithelial cells (Supplementary Fig. S3D). There were conserved epithelial alterations in female and male APP/PS1 mice, including downregulation of the polarity marker *Ezr* and upregulation of stress-responsive genes (e.g., *Apoe, Fcrla* and *Hes1*) although some difference occurred in mitochondria-associated dysfunctions or immune regulations. We also observed upregulation of lymphoid lineage markers (*Cd79a*, *Nkg7*, *Ptprap*, *Thy1*, *Cd3g*, *Trac*, and *Trbc2*) and downregulation of myeloid markers (*Lyz2*, *Aif1*, *Cd14*, *C1qc*, and *Pf4*) in female APP/PS1 mice relative to female WT mice (Supplementary Fig. S3E-S3F). Considering the fact that the total number of cells in the female groups is relatively small and there is a sex-specific molecular pattern [61], we restricted our subsequent analyses and experimental validates based on male animals only.

Taken together, the above results indicated a predominant dysfunction of epithelial cells in both male and female AD mice although the ChP DEGs showed some differences associated with gender. Such sex-specific heterogeneity underscores the necessity of gender-stratified therapeutic strategies for AD.

### Structural and functional epithelium-determined ChP impairments in APP/PS1 mice

**Fig. 3:**
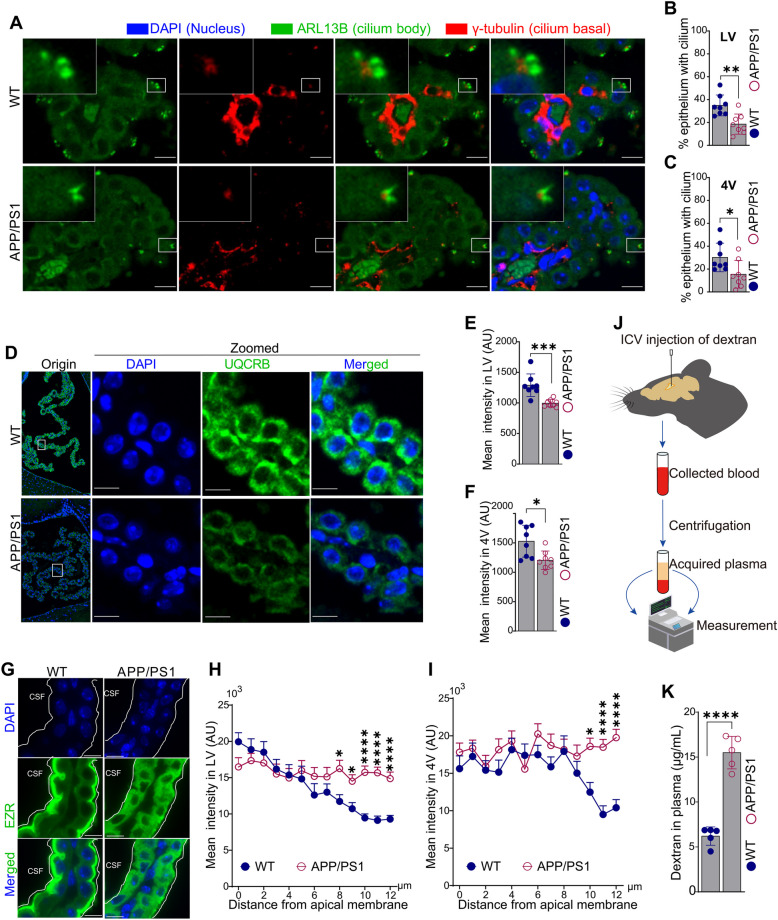
Structural and functional impairments of ChP epithelial cells in APP/PS1 mice. **A** Co-staining of ARL13B (green, cilium body) and γ-tubulin (red, cilium base) in APP/PS1 and wild-type (WT) mice. **B-C** Measurement of ARL13B niche numbers in ChP of the lateral (LV, **B**) and the fourth ventricles (4V, **C**) in (**A**). **D** UQCRB staining (green, a component of the mitochondrial respiratory complex III) of ChP in APP/PS1 and WT mice. **E–F** Measurement of fluorescence intensity in ChP of the lateral (LV, **E**) and the fourth ventricles (4V, **F**) in (**D**). **G-I** EZR staining (green, **G**) and measurement of fluorescence intensity distance from apical membrane (white dotted line) of epithelial cells in ChP of the lateral (LV, **H**) and the fourth ventricles (4V, **I**). **J** Workflow of permeability assessment experiment. **K** Comparison of dextran level in plasma after intracerebroventricular (ICV) injection in APP/PS1 and WT mice. Cell nuclei were stained by DAPI (blue). Scale bar, 10 μm. Histological validations (**A-I**; *n* = 8 per group) and dextran detection assays (**J-K**; *n* = 5 per group) were performed on independent age-matched male animals processed separately from those used for scRNA-seq. Statistical analyses in (**F-G**), (**I-J**) and (**K**) were performed using Student’s t-test and data were presented as mean ± SD; statistical analyses in (**H-I**) were performed using two-way ANOVA test and data were presented as mean ± SEM; *, *p* < 0.05; **, *p* < 0.01; ***, *p* < 0.001; ****, *p* < 0.0001

To validate the transcriptional changes of the ChP epithelium in male APP/PS1 mice, we further confirmed epithelial DEGs through immunostaining (Fig. 3A-I) and permeability assays (Fig. 3J-K). Assessment of cilium formation, by labeling the cilium body (ARL13B) and cilium base (γ-tubulin), demonstrated a reduction in the density of cilium units or niches in epithelial cells of 4-month-old APP/PS1 mice compared to that of WT mice (Fig. 3A-C). Of note, cilium-related genes were highly expressed in both epithelial and ependymal cells (Fig. 1E). While acetyl-tubulin staining detected cilium bodies in the ependymal cells, the cilia in ChP epithelial cells were shorter and more dispersed compared to those in ependymal cells (Supplementary Fig. S4). These observations suggested a tissue-specific, cilium-dependent physiological function in the ChP and ependymal cells.

Consistent with the transcriptomic alterations observed, the impaired assembly of the mitochondrial respirasome in the ChP of AD mice was corroborated by immunostaining for UQCRB, a component of the mitochondrial respirasome complex III [62]. Immunostaining revealed a marked reduction in UQCRB expression in the epithelial cells of the ChP in APP/PS1 mice (Fig. 3D-F). The integrity of interface between the CSF and blood relies on epithelial polarity and cell junction strength [20, 63, 64]. In our DEG analysis, genes associated with epithelial polarity (*Ezr*) and tight junctions (*Cldn10*) were down-regulated in the epithelial cells of the ChP in APP/PS1 mice (Fig. 2F). Immunostaining for EZR further revealed localization at the apical membrane (adjacent to the CSF) of epithelial cells in WT mice but decreased and redistributed expression in APP/PS1 mice, and quantification of EZR expression from the apical to basal membrane showed a more even distribution in APP/PS1 mice, indicating a loss of epithelial polarity in the ChP at the early stages of AD (Fig. 3G-I).

To further investigate whether the structural impairments in ChP epithelial cells disrupted the permeability barrier between the CSF and blood, dextran conjugated to a fluorophore was injected into the lateral ventricle, and its levels in plasma were measured (Fig. 3J). Indeed, a higher level of plasma dextran after intracerebroventricular injection was observed in APP/PS1 mice compared to control mice (Fig. 3K), indicating disruption of epithelial or ChP integrity at the early stages of AD.

### Down-regulation of epithelial MIF disrupts immune homeostasis, promoting pro-inflammation in brain of APP/PS1 mice

**Fig. 4:**
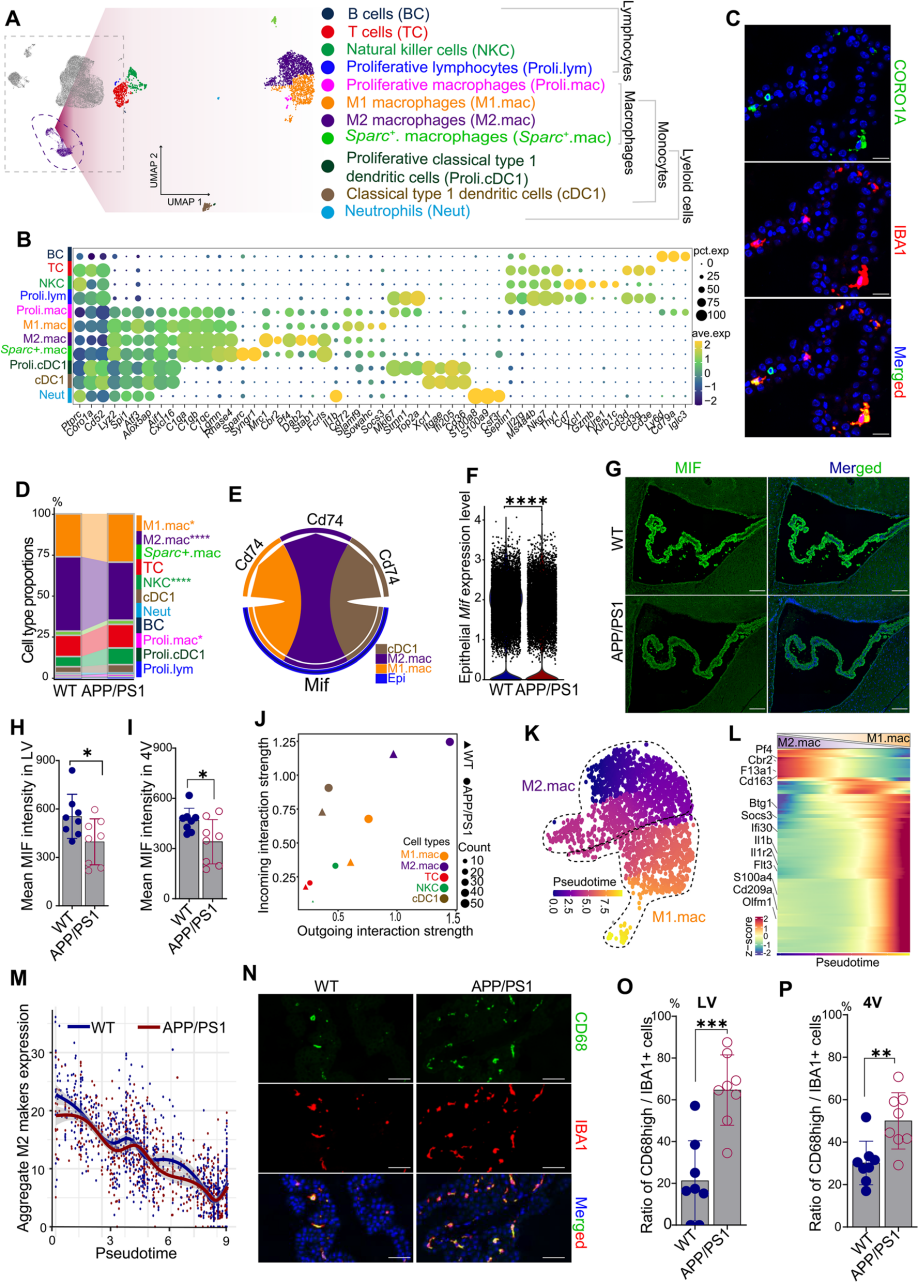
Down-regulation of epithelial MIF resulted in macrophages activation in APP/PS1 mice. **A** UMAP of immune subclusters in APP/PS1 and wild-type (WT) mice. **B** Subcluster markers and categorization of immune cells defined in (**A**). **C** Validation of immune cells and myeloid monocytes with co-staining of CORO1A (green, immune cells) and IBA1 (red, monocyte) in WT mice (*n* = 3). **D** Comparative analysis of cellular proportions across distinct cell types between APP/PS1 and WT mice. **E** MIF mediated ligand-receptor subcluster communications. **F** Comparison of epithelial *Mif* expression levels between APP/PS1 and WT mice. **G** MIF (green) in situ expression profiles in APP/PS1 and WT mice. Quantitative analysis of the lateral (LV, **H**) and the fourth ventricles (4V, **I**) in (**G**). **J** Comparison of immune subcluster interaction strength. **K** Pseudotime trajectory originating from M2 macrophages to M1 macrophages in UMAP. **L** Dynamic gene expression in the trajectory in (**K**). **M** Comparison of trajectory-associated expression levels of M2 markers in (**L**) along trajectory in APP/PS1 and WT mice. **N** Activated macrophages (M1) were validated by CD68 (green, classical marker for activated macrophages) and IBA1 (red) in ChP from APP/PS1 and WT mice. **O-P** Measurement on ChP in the lateral (LV, **O**) and the fourth ventricles (4V, **P**) in (**N**). Cell nuclei were stained by DAPI (blue). Scale bar, 20 μm. Histological validations (**G-I**, **N-P**) were performed on independent age-matched male animals (*n* = 8 per group) processed separately from those used for scRNA-seq. Statistical analyses in (**D**) were performed using Chi-square test. Statistical analyses in (**H-I**) and (**O-P**) were performed using Student’s t-test, and data were presented as mean ± SD. *, *p* < 0.05; **, *p* < 0.01; ***, *p* < 0.001; ****, *p* < 0.0001

Given that the ChP serves as a key immunological hub in response to acute brain inflammation [65] and that immune cells are predominant in the ChP stroma, a more detailed analysis of the immune cell population was conducted. This high-resolution dissection identified 8 distinct immune subclusters and 3 related proliferative subclusters (e.g., proliferative macrophages and proliferative lymphocytes) (Fig. 4A-B), which expressed common immune markers (e.g., *Coro1a*, *Ptprc*, and *Cd52*). The immune subclusters could be divided into myeloid (characterized by markers *Spi1*, *Lyz2,* and *Atf3*) and lymphoid lineages (characterized by specific markers *Septin1* and *Il2rg*). Approximately 76.6% of the immune cells belonged to the myeloid lineage, which contained monocytes (characterized by specific markers *Aif1*, *Cxcl16*, and *H2-Ab1*) and neutrophils (characterized by markers *S100a8* and *S100a9*) (Fig. 4A-B, Supplementary Fig. S5, and Supplementary Table S4). The monocytes were composed of macrophages (characterized by specific markers *C1qa*, *C1qb*, and *C1qc*), conventional type 1 dendritic cells (cDC1) (characterized by specific markers *Xcr1*, *Clec9a*, *Ifi205*, and *Cd36*), and proliferative cells (Proli) (characterized by specific markers *Mki67*, *Top2a*, *Stmn1*, and *Cdca8*). In contrast, the lymphoid lineage contained T cells (characterized by specific markers *Cd3d*, *Cd3e*, *Cd3g*, and *Trac*), natural killer cells (NKC) (characterized by specific markers *Cd7, Ncr1*, *Prf1*, and *Gzmb*), B cells (characterized by specific markers *Ly6d*, *Iglc3*, and *Cd79a*). Notably, proliferative cells exhibited high expression of proliferative markers and other cluster specific markers, suggesting a role in the clonal proliferation of these cell types (Fig. 4A-B and Supplementary Fig. S5).

To further understand the immune landscape in APP/PS1 mice, immune cells and monocytes were validated through in situ co-immunostaining for CORO1A and IBA1 (encoded by the *Aif1* gene) (Fig. 4C). To investigate immune homeostasis dysregulation in APP/PS1 mice, we compared intergroup proportions of immune cell subtypes. APP/PS1 mice exhibited a significant increase in pro-inflammatory M1 macrophages and NKC, with reduced M2 macrophages (Fig. 4D), indicating disrupted immune equilibrium and hyperactivation.

We further explored immune-stromal crosstalk in ChP. Cell–cell communication analysis revealed that epithelial cells interact with macrophages and cDC1 subsets predominantly mediated by MIF (Fig. 4E). Quantitative assessments demonstrated opposing regulatory patterns in APP/PS1 mice versus WT mice, with PF4-mediated signaling being significantly downregulated while APOE/APP-driven stress responses showed pathological over-activation in APP/PS1 mice (Supplementary Figs. S6A-S6B). Analysis of DEGs showed significant downregulation of MIF in ChP epithelial cells of APP/PS1 mice (Fig. 4F). Aggregated gene expression analysis confirmed epithelial cells as the primary source of ChP-derived MIF (Supplementary Fig. S6C). Immunohistochemistry with anti-MIF antibodies confirmed that MIF was predominantly localized to ChP epithelial cells, with minimal parenchymal expression (Fig. 4G), and protein-level quantification (Fig. 4H-I) corroborated the transcriptional finding. Enhanced cellular interactions within the ChP immune niche were observed in APP/PS1 mice, involving M1/M2 macrophages, NKC cells, and cDC1 populations (Fig. 4J). These results suggested that reduced epithelial MIF expression may alleviate immune suppression and trigger pathological activation. Pseudotime trajectory analysis revealed accelerated M2-to-M1 macrophage differentiation in APP/PS1 mice, evidenced by dynamic upregulation of Interleukin-1β (IL1β, terminal M1 marker) (Fig. 4K-M and Supplementary Fig. S6D). To confirm the activation of proinflammatory macrophages, CD68 (a well-known marker for M1 macrophages [33, 66, 67]) was labeled and found to be significantly up-regulated in the ChP of APP/PS1 mice compared to WT controls (CD68^high^IBA1^+^ cells) (Fig. 4N-P).

To further dissect the functional role of MIF in M1 polarization, we tested the effects of MIF inhibition on inflammatory status of macrophages using BMDMs from 4-month-old male WT mice (Supplementary Fig. S6E). BMDMs challenged by oligomeric Aβ showed an increase of TNFα secretion in an Aβ dose-dependent manner, and MIF inhibition by selective inhibitor 4-iodo-6-phenylpyrimidine (4-IPP) exacerbated Aβ-induced TNFα upregulation (Supplementary Figs. S6F-S6G). Strikingly, while IL1β remained unchanged under Aβ-only conditions at different concentrations, LPS priming activated IL1β, and MIF blockade combined with LPS priming synergistically enhanced IL1β expression (Supplementary Fig. S6H). With MIF blockade, oligomeric Aβ challenge potentiated the activation of IL1β in BMDMs (Supplementary Fig. S6H). These data collectively demonstrated that ChP epithelial MIF deficiency enables Aβ-dependent hyperactivation of M1 polarization, suggesting a ChP epithelial-immune axis in AD pathogenesis.

### ChP-ependyma interaction mediates lipid delivery and microglial activation in the parenchyma

**Fig. 5:**
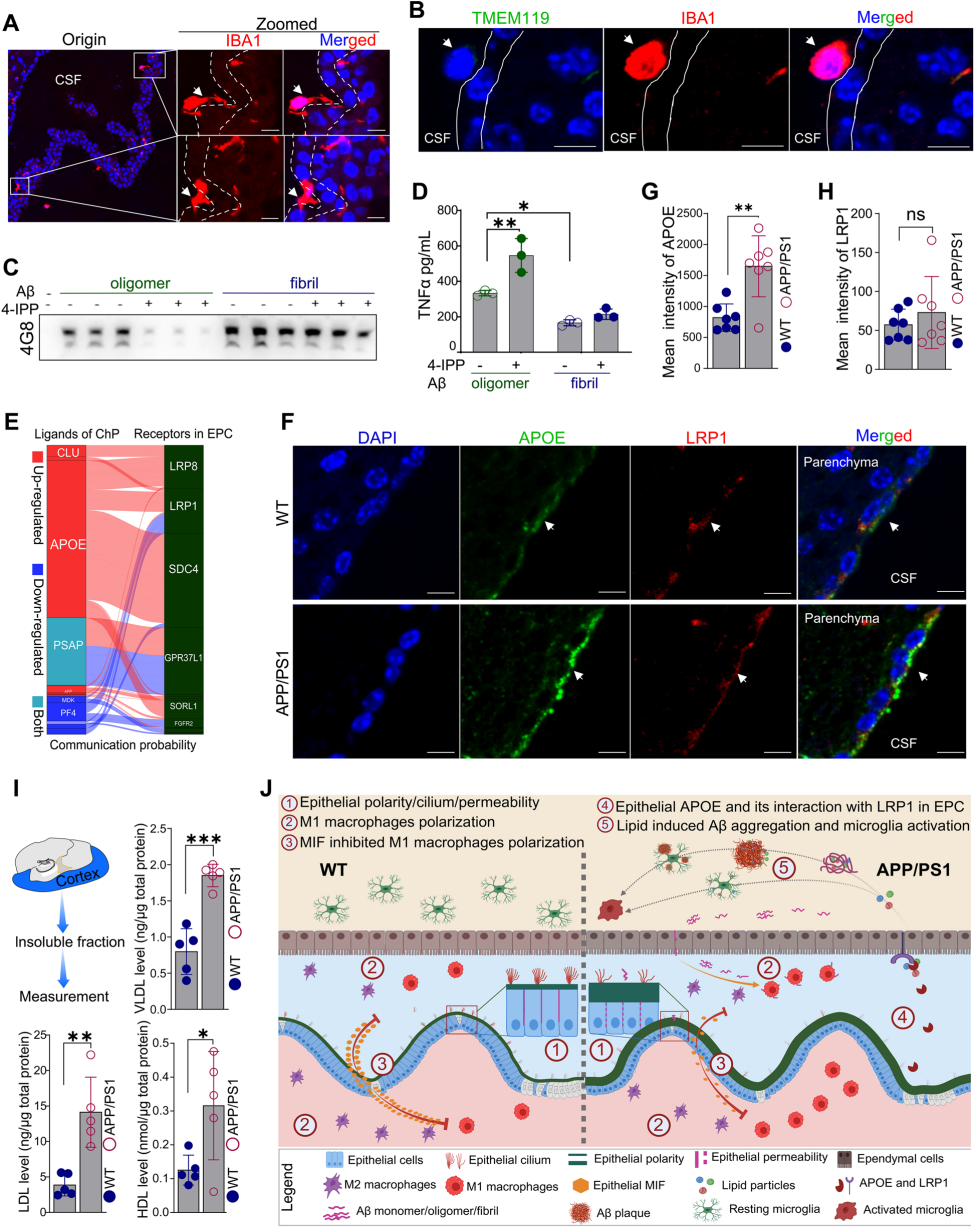
Increased APOE of ChP altered brain lipid homeostasis via interactions with receptors in ependymal cells. **A** Detection of monocytes (positive for IBA1, red) in CSF-ChP interface in APP/PS1 mice (*n* = 3). **B** Monocytes stained positive for IBA1 (red) but not TMEM119 (green) were detected in ependymal cells (white dotted line) in ChP from the APP/PS1 mice (*n* = 3). **C** Western blotting of the retained Aβ in culture medium of BMDMs from 4-month-old male WT mice (*n* = 3). **D** Quantification of TNF-α in supernatants from BMDMs culture in (**C**) using ELISA. **E** Up-regulated (red) and down-regulated ligands (blue) from ChP interact with their receptors in ependymal cells (green). **F** Co-staining of APOE (green) and LRP1 (red) in brain slice of APP/PS1 and WT mice. **G-H** Quantification of APOE (**G**) and LRP1 (**H**) fluorescence intensity in ChP of APP/PS1 (*n* = 7) and WT (*n* = 7) mice in (**F**). **I** Measurement of lipoprotein particles in insoluble fraction of cortex and hippocampus tissues from APP/PS1 (*n* = 5) and WT (*n* = 5) mice by using ELISA. HDL, high-density lipoprotein; LDL, low-density lipoprotein; VLDL, very low-density lipoprotein. **J** Working model showing pathological abnormalities in ChP of mice at the early stage of AD pathology. The image was created by Biorender. Cell nuclei in (**A**, **B** and **F**) were stained by DAPI (blue). Histological validations (**A-B, F-H**) and lipid particles measurement assay (**I**) were performed on independent age-matched male animals processed separately from those used for scRNA-seq. Scale bar, 20 μm. Data in (**D**), (**G-H**), and (**I**) were presented as mean ± SD, and statistical analyses were performed using Student’s t-test; ns, not significant; *, *p* < 0.05; **, *p* < 0.01; ***, *p* < 0.001

We investigated how activated macrophages contribute to the development of AD by measuring macrophage migration and phagocytosis. Through IBA1 immunostaining, we observed macrophages actively traversing the ChP, with robust signals at the CSF-ChP and CSF-ependymal interfaces (Fig. 5A-B), indicating that these immune cells can cross ChP barriers to exert regulatory functions within CSF or ependymal regions. To investigate Aβ phagocytic specificity, we treated BMDMs with Aβ monomers, oligomers, or fibrils. Detection of retained Aβ level in culture medium demonstrated preferential uptake of Aβ oligomers, and MIF inhibition significantly enhanced phagocytic activity (Fig. 5C and Supplementary Fig. S7A), suggesting that ChP macrophages directly participate in CSF Aβ clearance. Additionally, inhibition of MIF also enhanced the Aβ-induced TNFα expression levels (Fig. 5D), indicating the synergistic effects of MIF on macrophage-mediated Aβ phagocytosis and inflammation. These results suggested that manipulating epithelial MIF in ChP would be beneficial as a potential therapeutic approach. In addition to infiltration and phagocytosis, macrophage-ependymal cell communication analysis revealed abnormalities in ligands secreted by ChP cell types (especially macrophages), such as the up-regulation of APOE or CLU and down-regulation of PF4 (Fig. 5E), and increased macrophages-dominated communications in APP/PS1 mice (Supplementary Fig. S7B). Reanalysis of CSF proteomics from Bader et al. [12] confirmed high intensity of APOE and MIF levels (Supplementary Fig. S7C), suggesting that they may be mainly derived from ChP. Co-immunostaining further demonstrated that APOE co-localized with its receptor LRP1 in ependymal cells, with APOE expression but not LRP1 expression showing consistent up-regulation in the ChP of APP/PS1 mice (Fig. 5F-H). Analysis based on public source [59] revealed that the mRNA expression level of *Apoe* was higher in ChP than in cortex and hippocampus (Supplementary Fig. S7D). This indicated that APOE in ependymal cell might be mainly derived from ChP. All these observations suggested that targeting APOE or other genes in the ChP could influence ependymal cell function. Additionally, receptors involved in the dysfunctional communication between the ChP and ependymal cells were associated with binding of lipoprotein particles, including LRP1, LRP8, and SDC4, indicating potential disruptions in lipid transport within the parenchyma. This was supported by observed increases in HDL, LDL, and VLDL in the insoluble fraction of the brain cortex (Fig. 5I), which may contribute to plaque deposition and microglial activation in the cortex of APP/PS1 mice (Supplementary Figs. S7E-S7G), establishing a spatial link between ChP signaling and parenchymal pathology. This speculation was consistent with a previous report for an active involvement of lipid droplet accumulation in microglia in aging brain [68]. Note that the number of ependymal cells in our dataset might be too small for a reliable cell–cell communication, and we could not exclude a possibility for the presence of ependymal cells due to imperfect isolation of ChP tissue. Nevertheless, the identified ependymal-associated cell–cell interactions mediated by classical receptor-ligand pairs such as APOE-LRP1, have been experimentally validated in previous studies [69].

A working model of ChP dysfunction in AD development is thus proposed based on the above findings, redefining the ChP as a hub orchestrating AD pathogenesis (Fig. 5J). Single-cell transcriptomics combined with functional validation revealed that ChP epithelial cells in APP/PS1 mice underwent a population collapse, accompanied by disrupted cilium and mitochondrial assembly impairment, and compromised barrier integrity and permeability. Crucially, epithelial-derived MIF served as an immune checkpoint, whose downregulation triggered or licensed M1 macrophage polarization. These activated ChP macrophages transmigrate into CSF, synergizing with CSF-resident counterparts to enhance Aβ42 phagocytosis, yet paradoxically promoted inflammation in ChP and parenchyma. ChP derived APOE hypersecretion may interact with ependymal LRP1/LRP8/SDC4 receptors and drive cortical lipoprotein dysregulation. This lipid storm may accelerate Aβ aggregation and induced microglial activation. Our model positions the ChP as a regulator of structural integrity and immune surveillance, and pioneers a systems biology framework integrating choroidal immunity, lipid metabolism, and neuroinflammation into a unified pathophysiological axis for AD development.

## Discussion

The ChP plays a vital role in maintaining brain homeostasis, with accumulating evidence highlighting its involvement in AD [23, 24, 26, 28, 34]. Although previous studies have identified transcriptomic abnormalities in AD [26, 27, 70], a detailed understanding of these changes at the single-cell level is lacking. Single-cell analyses of ChP [65], which offer more granular insights than single-nucleus profiling [35, 71], were employed in this study to perform the first single-cell transcriptional analysis of the ChP in the context of AD. Six broad cell types were identified in ChP, allowing for comparison of cell type-specific transcriptional and cellular alterations in APP/PS1 mice. Notably, over 78% of DEGs were detected in epithelial cells, the predominant cell type in the ChP. These epithelial cells exhibited dysfunctions in several key physiological processes, including cilium assembly, mitochondrial respiratory complex formation, secretion, transport mechanisms, and interface integrity, which are fundamental to the proper functioning of the ChP in brain physiology [20, 72].

The ChP, situated at the critical junction between blood and CSF [20], is responsive to signals from both peripheral and central sources [73–75], particularly during brain inflammatory conditions [65, 76]. Neuroinflammation and immune activation are hallmark features of AD [45, 77], with the ChP serving as a key site for immune activity in response to brain inflammation [65]. In this study, we identified significant epithelial responses to stress in AD, likely triggered by signals from the brain parenchyma, such as the Aβ fraction and cytokines [11, 14, 78, 79]. Emerging evidence supports the role of the ChP as an immune checkpoint, actively participating in the response to inflammation or infection [26, 33, 65, 71, 75, 80, 81]. The ChP hosts a diverse array of immune cells, similar to those found in other brain barriers or interfaces, such as the dura, leptomeninges, and perivascular spaces [38, 82]. The recruitment of monocytes to the ChP following intracerebroventricular injection of LPS [65] aligns with the immune response observed in AD [31]. To further elucidate the immune landscape in the ChP of APP/PS1 mice, 11 distinct immune subclusters were defined by scRNA-seq, revealing transcriptional abnormalities that closely resemble the immune cell populations identified by CD45 positive cells [38]. Notably, M2 macrophage dysfunction emerged as a key factor in immune activation, potentially due to decreased levels of epithelial MIF [83, 84]. Additionally, a novel proliferative immune cell type was increased in APP/PS1 mice, suggesting that the increase of M1 macrophages may be driven primarily by resident clonal proliferation rather than peripheral recruitment [85]. Given the limited number of proliferative cells identified, further research with larger datasets is necessary to fully elucidate the mechanism and implications of these findings.

Monocytes, predominantly composed of macrophages, were detected in proximity to CSF and ependymal cells, suggesting potential crosstalk between macrophages and both the epithelium and ependyma. An increase in monocyte populations within the ChP was noted, with subsequent entry into the brain parenchyma to regulate amyloid pathology and activate microglia in the context of AD [31] and infection [65]. Macrophages in ChP exhibited distinct disease-associated microglia-like phenotypic features compared to parenchymal microglia. These cells were characterized by significantly upregulated expression of lipid metabolism-related molecules (e.g., *Apoe*) and immune activation markers (e.g., *Il1b*, *Csf1* and *Cst7*) [38], indicating that ChP maintained a stronger baseline inflammatory state than the brain parenchyma under homeostasis. Under physiological conditions, the ChP barrier system (including tight junction proteins CLDN1 or CLDN5) strictly restricts the infiltration of activated macrophages into the parenchyma. However, peripheral inflammation [75], aging [86], or AD pathology [87] could disrupt this barrier integrity, allowing activated macrophages in APP/PS1 mice [31] or infection-induced inflammatory cells [36] to breach the barrier and infiltrate the parenchyma, and exacerbate neuroinflammatory cascades. Our findings demonstrated that permeability defects and polarity impairment in the ChP emerge during a relatively early age with less Aβ pathology, suggesting these structural and functional ChP alterations may serve as initiating factors that accelerate AD progression. The ChP-barrier dysfunction in AD mouse models shared phenotypic similarities with aging-related pathology in junctional components (e.g., CLDN1 and ICAM1) [35, 86] and immune mediators (e.g., IFN-γ) [88]. The ChP structural deficits across age groups might reflect progressive severity escalation rather than divergent pathological trajectories or types. Notably, M2 macrophages were found to be the major cell type dysregulating ependymal cell function via the secretion of ligands such as APOE, facilitating lipid entry into the parenchyma under the control of receptors in ependymal cells. The accumulation of lipids, particularly in a proinflammatory state, has been linked to microglial activation during normal aging at late life [68]. APOE4 knock-in mice and AD patients exhibited substantial lipid droplet accumulation in ChP [33], where high expression levels of lipid transporting associated proteins (e.g., *Apoe*, *Clu* and *Lrp1*) implied that lipid dysregulation in the brain parenchyma of AD patients may be attributed to impaired lipid-processing specialization of this compartment. Moreover, the presence of monocytes within ependymal cells, along with the up-regulation of APOE and down-regulation of PF4 in the ChP, highlighted the role of ependymal cells as the coordinators responding to ChP stress, ultimately resulting in microglial activation in the brain parenchyma. These findings collectively demonstrated that APOE transcends its classical role as an immune checkpoint mediator in ChP [33] to establish a dual-compartment regulatory paradigm governing both ChP-mediated immunosurveillance and parenchymal immunomodulation. This conceptual expansion substantially advances our mechanistic understanding of the role of APOE in AD pathogenesis. Nevertheless, it should be emphasized that while APOE demonstrated polarized enrichment on the CSF-facing ependymal surface, exhibited high abundance in CSF, and showed an elevated mRNA expression in ChP compared to hippocampal and cortical regions, contributions from parenchymal APOE pools [89] interacting with ependymal LRP1 could not be definitively excluded. Future studies with ChP-specific APOE rescue based on systemic APOE knockout models will be essential to determine the exact effect of ChP-derived APOE on ependymal and parenchymal functions.

Although this study identified novel abnormalities in the ChP at the early stage of AD, offering valuable insights into the pathobiology of AD, several limitations should be addressed. First, the direction of cell proportion changes between females of APP/PS1 and WT was opposite to that of males, potentially due to a higher proportion of epithelial cells in female mice (Supplementary Fig. S3). This finding was consistent with a report for a larger ChP in females than in males [90]. We determined intrinsic sex differences within the same genotype (Supplementary Figs. S8A-B), and these differences intersected with DEGs between APP/PS1 and WT mice in same sex (Supplementary Figs. S8C-S8F), suggesting that the sex-biased transcriptional profiles profoundly influenced DEGs between APP/PS1 and WT mice. These results indicated sex-dependent heterogeneity in ChP pathology during early AD progression, which was consistent with reported sex effects in ChP [90–92] and other tissues [61, 93, 94]. However, due to the limited number of pooled samples (two male pools and one female pool), these findings should be interpreted with caution, and further validation with larger cohorts is warranted. Second, the ChP plays a crucial role in secreting molecules into the CSF to modulate nervous system function, including serotonin [95], insulin [96, 97], ions [80, 98], and microRNAs [99]. However, whether the observed cellular alterations in the ChP during AD development affect the production of these molecules, or whether mutual communication exists among them, remains to be determined. The ChP functions not merely as a structural component of the blood-CSF barrier maintaining neurochemical homeostasis but as a master regulatory interface coordinating neuroprotection through CSF volumetric regulation, metabolic waste clearance, and neurotrophic factor secretion. Considering that the entire nervous system is immersed in CSF [20], a tripartite investigative framework simultaneously addressing ChP pathophysiology, CSF composition dynamics, and parenchymal network remodeling is helpful to establish holistic neuropathological signatures. Moreover, a combined analysis of parenchyma profiling at the single-cell level with ChP data would provide more insight into the question regarding the immune and lipid dysregulation occur earlier or later or at the same time in the ChP compared to parenchyma. Third, the alterations of ChP during aging [35, 86] and the chronic neurodegenerative nature of AD further underscore the translational imperative for longitudinal multi-parametric profiling of ChP phenotypic evolution across different disease stages, coupled with mechanistic investigation of ChP-parenchymal crosstalk. It would have been interesting to have another timepoint later in the course of pathology to see if the changes observed at the early stage continue in the same direction, or other types of dysfunctions appear. Moreover, we should perform mechanistic experiments to understand if the changes observed are playing a driving role, or are just consequences of elevated Aβ production, or if it is a circular loop whereby the ChP dysfunction worsens the amyloid pathology. Such investigations could illuminate temporal progression patterns of ChP immune dysregulation, trans-compartmental propagation kinetics of pathobiological mediators, and therapeutic viability of ChP-targeted early interventions of parenchymal function. Future basic and clinical studies implementing synchronized serial CSF-ChP-parenchyma aligned with clinical disease staging, leveraging spatial multi-omics platforms preserving neuroanatomical context, would very likely providing new insights for AD management.

## Conclusions

In summary, this study characterized the cellular alterations within the ChP to better understand its role in the progression of AD pathology. Gaining deeper insights into these transcriptional and cellular changes may provide novel perspectives on the underlying pathobiology and potential treatments for AD. Developing new approaches and tools for manipulating the ChP will be crucial for accelerating research into the characteristics and changes of the ChP during AD progression.

## Supplementary Information

Supplementary Material 1.

## Data Availability

The raw sequencing data (fastq format) were deposited in the China National Center for Bioinformatics (CNCB) under accession number PRJCA028600. All analyses were performed using freely available software packages. Custom code from the current study is available from the authors upon reasonable request.

## Abbreviations

AD: Alzheimer disease
ChP: Choroid plexus
BCSFB: Blood-cerebrospinal fluid -brain barrier
CSF: Cerebrospinal fluid
scRNA-seq: Single cell RNA sequencing
Aβ: Amyloid-β
APP: Amyloid-β precursor protein
APOE: Apolipoprotein E
CLU: Clusterin
DEG: Differentially expressed genes
UMAP: Uniform Manifold Approximation and Projection
SD: Standard deviation
SEM: Standard error of the mean
TSA: Tyramide signal amplification
HRP: Horseradish peroxidase
DAPI: 4′,6-Diamidino-2-phenylindoe
OTX2: Orthodenticle homeobox 2
ACTA2: Actin alpha 2 or smooth muscle
PECAM1: Platelet/endothelial cell adhesion molecule 1
PF4: Platelet factor 4
MHC II: Major histocompatibility complex class II
EZR: Ezrin
UQCRB: Ubiquinol-cytochrome c reductase binding protein
ARL13B: ADP-ribosylation factor-like 13B
Ac-Tubulin: Lys40-Acetyl-Tubulin
CORO1 A: Coronin, actin binding protein 1A
CD68: CD68 antigen
Aif1: Allograft inflammatory factor 1
IBA1: Ionized calcium binding adaptor molecule 1
MIF: Macrophage migration inhibitory factor
LRP1: Low density lipoprotein receptor-related protein 1
HDL: High density lipoprotein
LDL: Low density lipoprotein
VLDL: Very low density lipoprotein
