# Supplementary material for: Early transcriptional and cellular abnormalities in choroid plexus of a mouse model of Alzheimer’s disease
Source: Mol Neurodegener. 2025 May 31;20:62. doi: 10.1186/s13024-025-00853-w (PMC12125878; doi:10.1186/s13024-025-00853-w)
Supplement: Supplementary file 1 — Supplementary Material 1. [file 13024_2025_853_MOESM1_ESM.zip › Supplementary information.docx]

Online Supplementary Materials

**Early transcriptional and cellular abnormalities in choroid plexus of a mouse model of Alzheimer’s disease**

Zhong-Jiang Yan^1, 2, *^, Maosen Ye^1, *^, Jiexi Li^1, 2^, Deng-Feng Zhang^1, 2, 3, #^,Yong-Gang Yao^1, 2, 3, #^

This DOC file includes:

Supplementary Figures S1-S8 and legends

Supplementary Tables 1-4


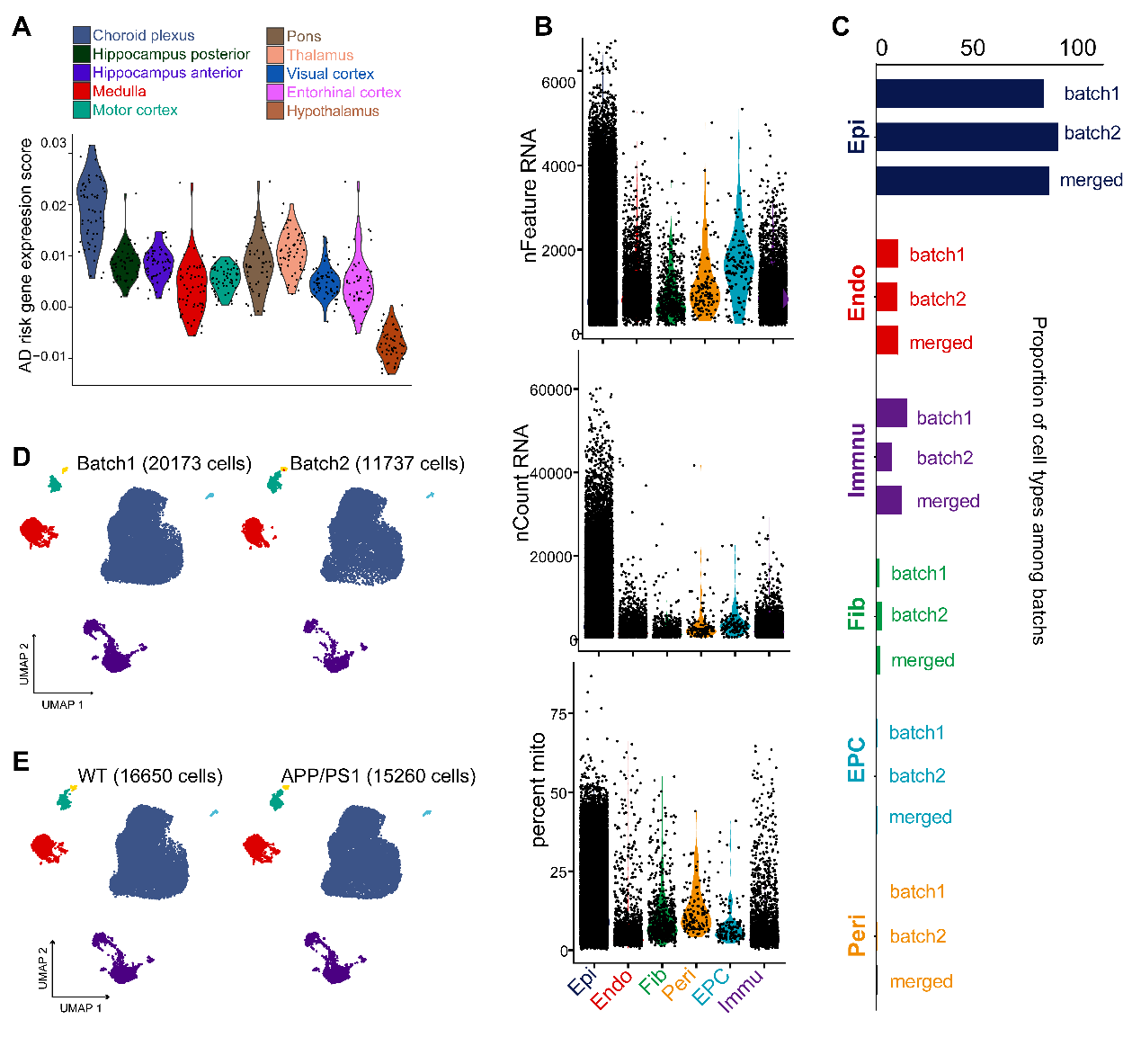


**Supplementary Figure S1. Expression of AD-associated genes in different brain tissues in AD patients and scRNA-seq analysis of the choroid plexus (ChP) from APP/PS1 and wild-type (WT) mice.**

(**A**) AD-associated gene enrichment scores in ChP and different brain regions from patients with AD. The AD-associated gene enrichment scores (normalized gene set expression level) were calculated using the *AddModuleScore_UCell* function in the *UCell* R package based on AD risk genes near AD risk loci [1] and AD causative genes, including *APP*, *PSEN1*, and *PSEN2* [2]. Original transcriptomic dataset was taken from Hahn et al. [3].

(**B**) Number of genes (nFeature_RNA), mRNA molecules (nCount_RNA), and percentage of mitochondrial genes (percent.mito) in all ChP cells from APP/PS1 and WT mice after scRNA-seq data quality control.

(**C-D**) Inter-batch comparative analysis of cell type proportions following dimensionality reduction and clustering. (**C**) Comparative analysis of cellular composition heterogeneity between Batch 1 and Batch 2. Results in the main text were based on combined batches. (**D**) Comparative UMAP visualization of ChP cell types across Batch 1 and Batch 2.

(**E**) Comparison of UMAP between APP/PS1 and WT mice. Each group has 8 animals.

Epi, epithelial cells; Endo, endothelial cells; Immu, immune cells; Fib, fibroblasts; Peri, pericytes; EPC, ependymal cells.


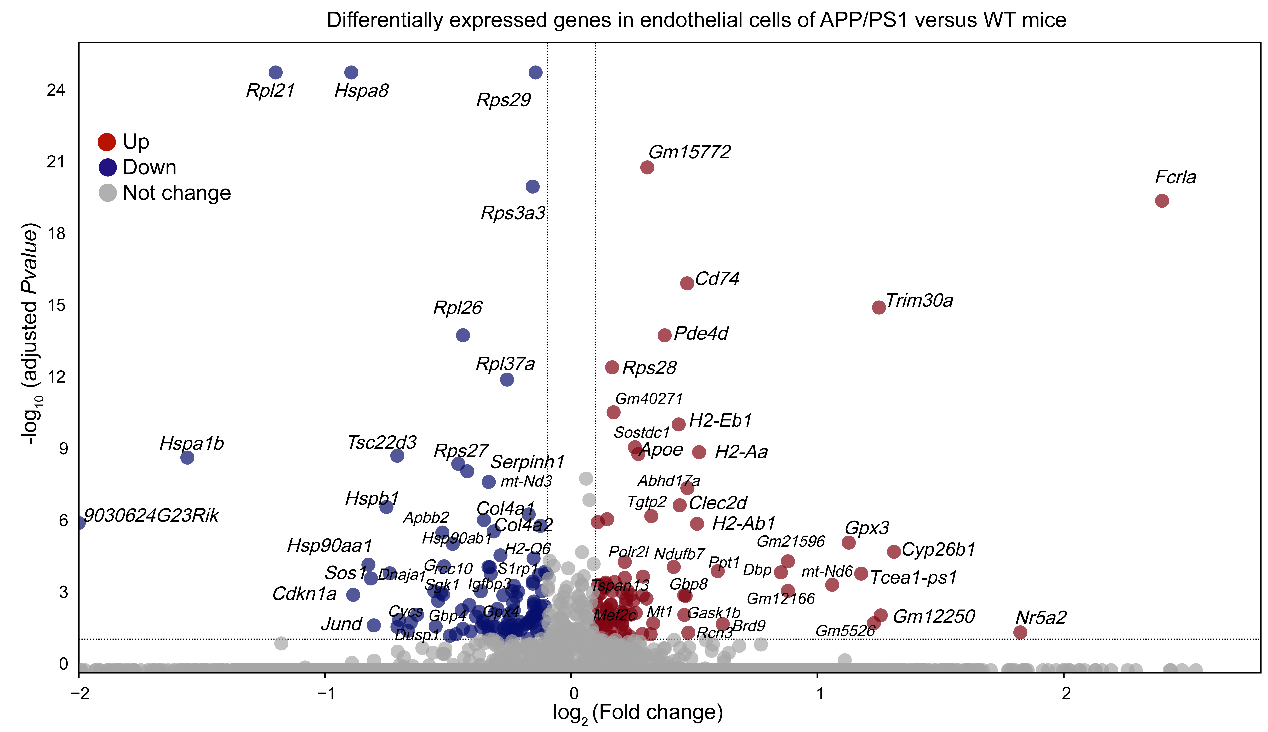


**Supplementary Figure S2.** **Volcano plot of endothelial DEGs between male APP/PS1 and wild-type (WT) mice (n = 8 per group).**


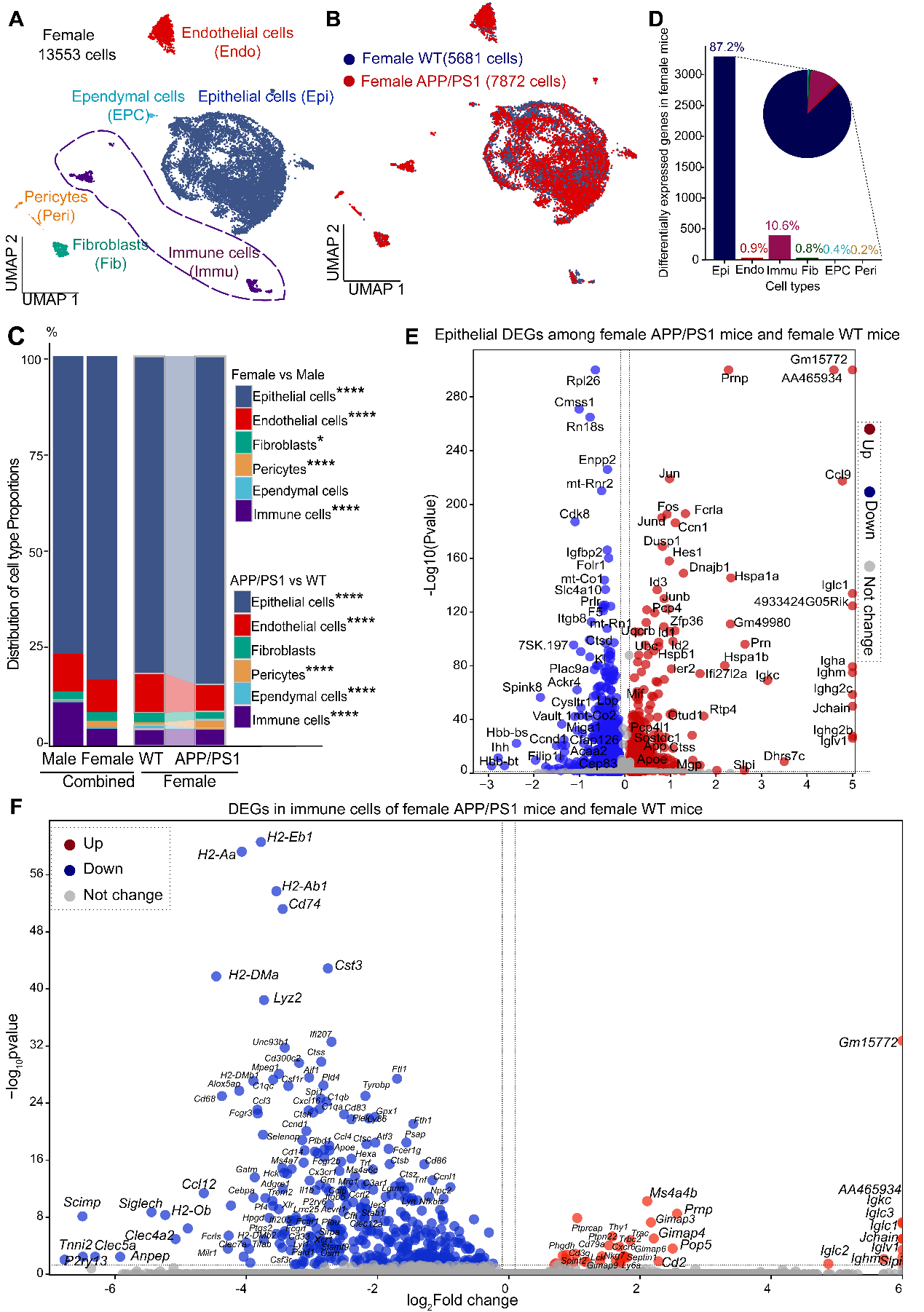


**Supplementary Figure S3. Transcriptomic cellular and gene expression changes in female APP/PS1 and female wild-type (WT) mice with early-stage Aβ pathology.**

(**A**) UMAP plot of cell types in ChP of female mice. The ChP tissues from three female mice per group were pooled together as one sample for scRNA-seq.

(**B**) Comparison of UMAP clustering distribution between female WT and APP/PS1 mice.

(**C**) Differences in cell proportions between female and male mice, and between female APP/PS1 and female WT mice. Statistical significance was analyzed using the chi-square test, **, *P* < 0.05; ****, *P* < 0.0001.

(**D**) Comparison of the number of differentially expressed genes (DEGs) between female APP/PS1 and WT mice across cell types.

(**E-F**) Volcano plots of DEGs in epithelial cells (**E**) and in immune cells (**F**) from female APP/PS1 and WT mice.


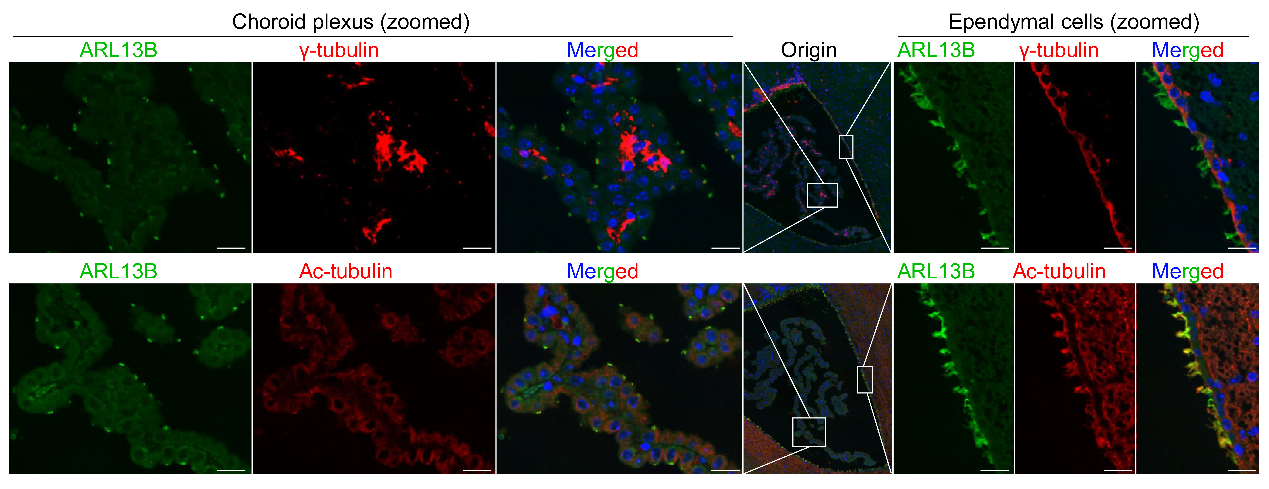


**Supplementary Figure S4. Cilium morphology in epithelial and ependymal cells.** Cilium bodies were stained by ARL13B (green) or Ac-tubulin (red); cilium bases were stained by γ- tubulin (red); nuclei were stained by DAPI (blue). 4-month-old male WT mice (n = 3) were used for this analysis. Scale bar, 50 μm.


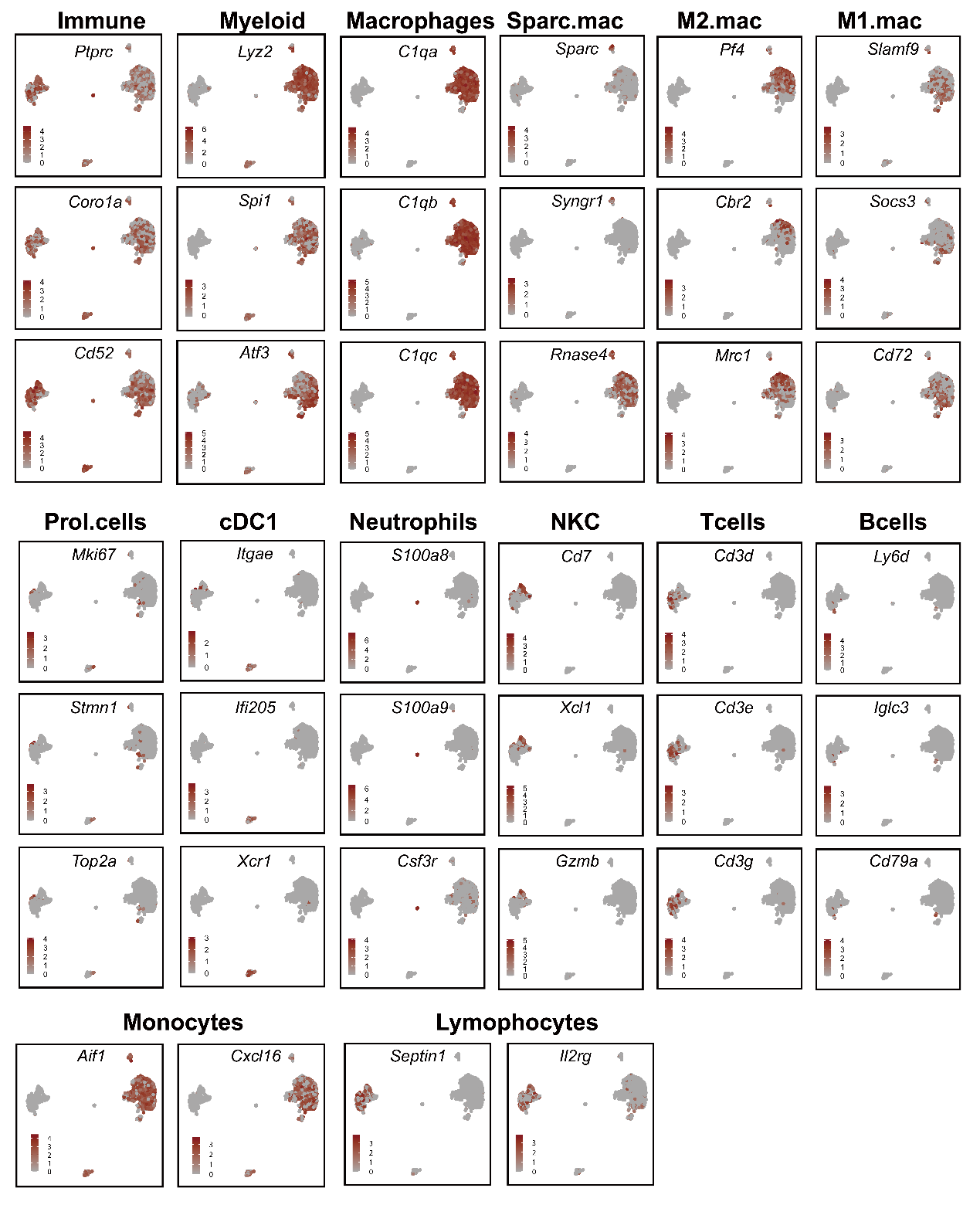


**Supplementary Figure S5. Expression level of immune subcluster marker genes in UMAP.** The UMAP was based on single cells from pooled ChP samples from APP/PS1 and WT mice (n = 8 per group).


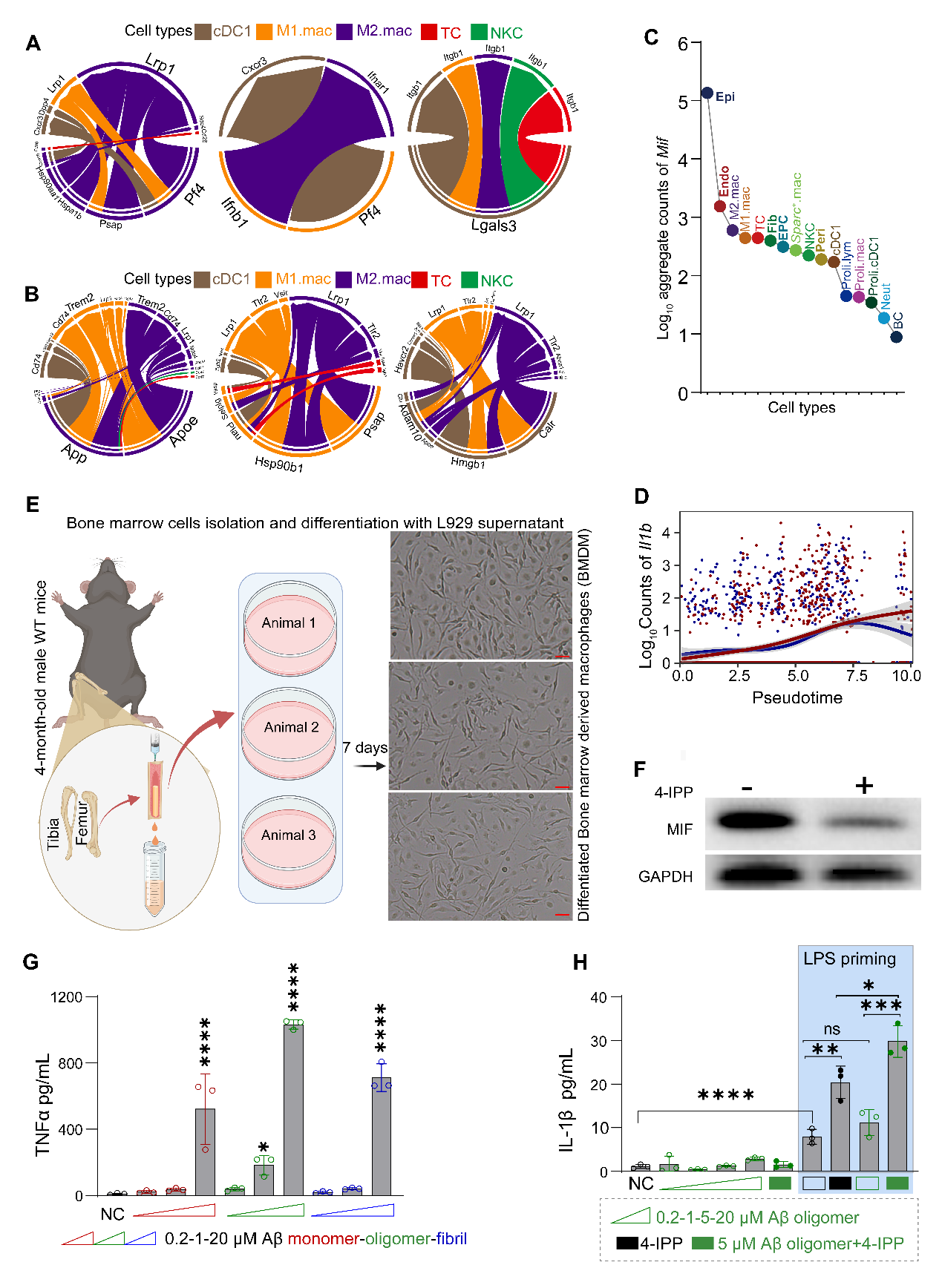


**Supplementary Figure S6. Cell communications in immune subclusters between APP/PS1 and WT mice and experimental characterization of bone marrow derived macrophages (BMDMs) from WT mice.**

(**A-B**) Downregulated (**A**) and upregulated (**B**) communications of M2 macrophages, M1 macrophages and cDC1 between APP/PS1 and WT mice.

(**C**) Aggregate expression level of *Mif* in distinct cell types of the ChP from APP/PS1 and WT mice.

(**D**) Expression level of *Il1b* (marker of M1 macrophages) associated with trajectory.

(**E**) Workflow for BMDMs isolation and characterization. Bar, 10 μm.

(**F**) MIF expression in BMDMs with and without stimulation by 4-IPP. Cells treated by 4-IPP (40 µM) for 48 h before harvest for Western blotting.

(**G**-**H**) Quantification of TNF-α (**G**) and IL-1β (**H**) in supernatants from BMDMs culture using ELISA. Each group has three biological replicates (n = 3). 4-IPP, MIF inhibitor. Data are expressed as mean ± SD. For only Aβ monomers, Aβ oligomers and Aβ fibrils (at varying concentrations) and LPS-treated groups comparisons with the NC, were performed using one-way ANOVA. LPS-primed groups were analyzed by one-way ANOVA with pairwise comparisons. *p* < 0.05; **, *p* < 0.01; ***, *p* < 0.001; ****, *p* < 0.0001.


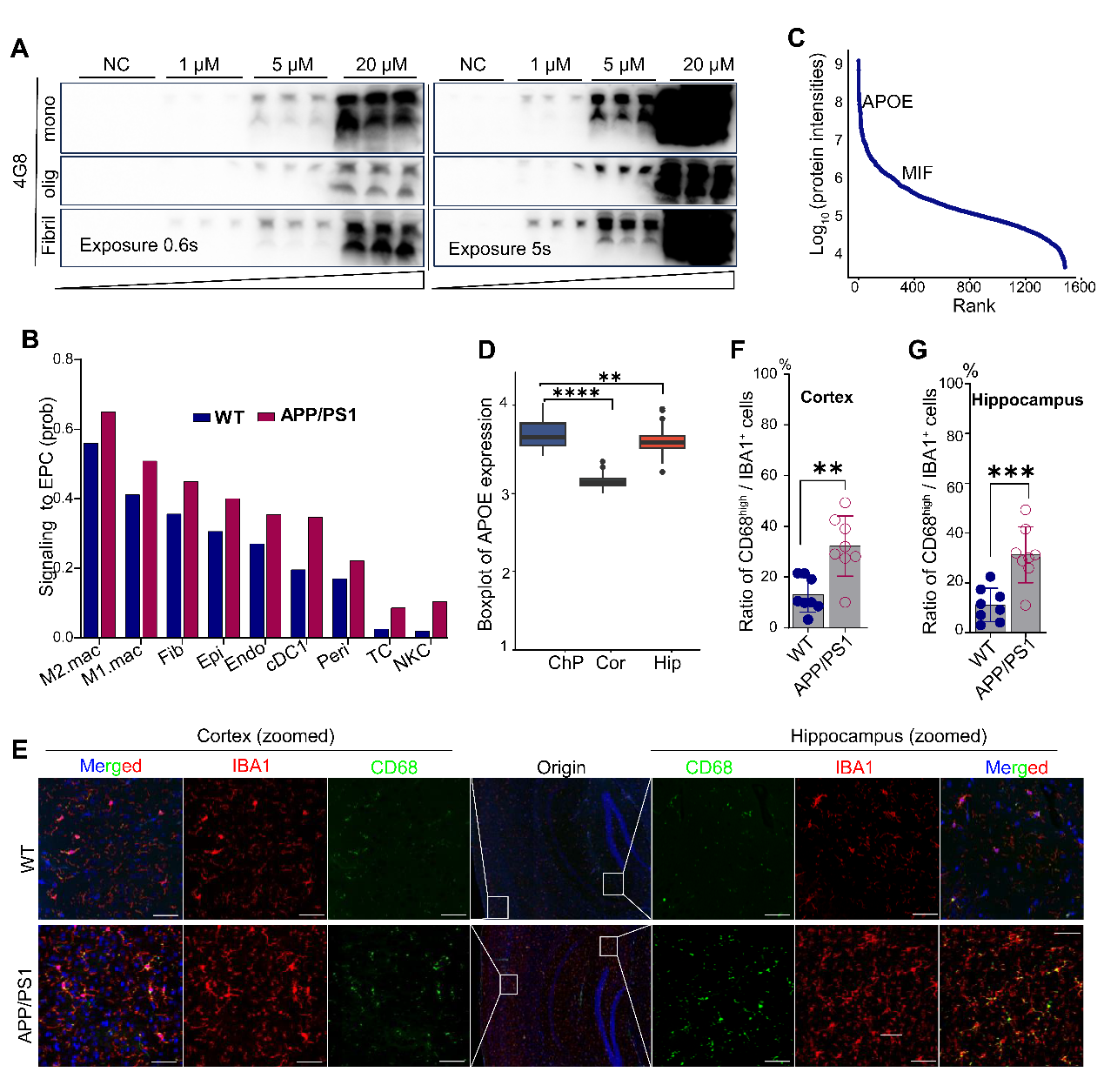


**Supplementary Figure S7. Ependymal cell signaling and microglial activation in cortex and hippocampus of APP/PS1 mice.**

(**A**) The level of Aβ in culture medium of BMDMs from WT mice detected by Western blotting for 4G8 (n =3 biological replicates per group).

(**B**) Communication strength between different ChP cell types and ependymal cells in APP/PS1 and WT mice, with cell types categorized using black lines.M2.mac, M2 macrophages; M1.mac, M1 macrophages; Fib, fibroblasts; Epi, epithelial cells; Endo, endothelial cells; cDC1, conventional type 1 dendritic cells; Peri, pericytes; TC, T cells; NKC, natural killer cells.

(**C**) Protein intensities in CSF. Data were derived from Bader et al. [4].

(**D**) Normalized expression counts of *APOE* in different brain tissues from AD patients. ChP, choroid plexus; Cor, cortex; Hip, hippocampus. The original data was taken from Schwartzentruber et al. [1].

(**E**) Co-staining of CD68 (green) and IBA1 (red) in cortex and hippocampus of APP/PS1 and WT mice, with nuclei stained by DAPI (blue).

(**F-G**) Proportion of activated microglia (CD68^high^ IBA1^+^ cells) in cortex (**F**) and hippocampus (**G**) from (**E**). Histological validations were performed on independent age-matched male cohorts (n = 8 per group) processed separately from those used for scRNA-seq. Scale bar, 50 μm. Data are presented as mean ± SD. Statistical analyses were performed using Student's t-test; **, *p* < 0.01; ***, *p* < 0.001.


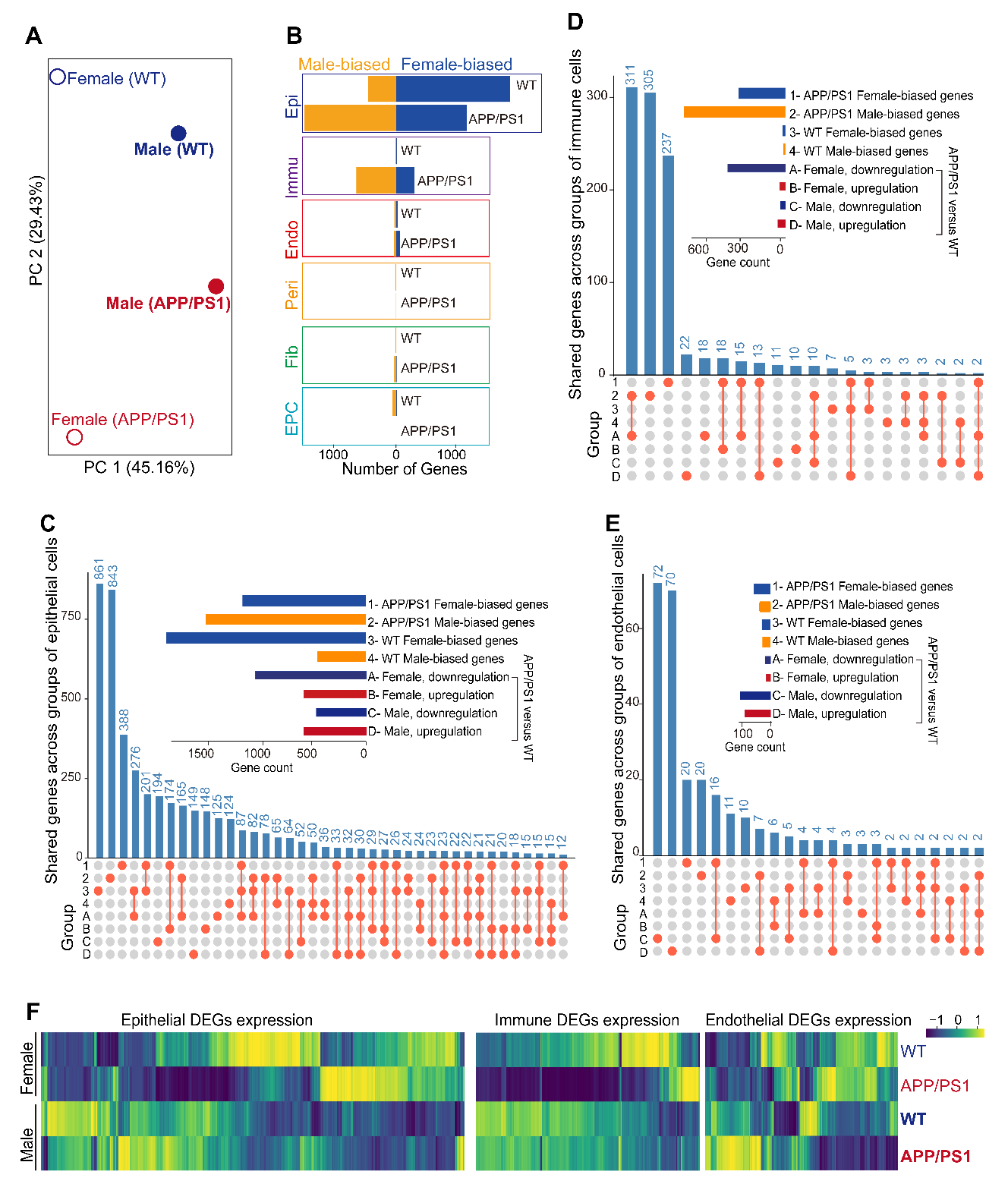


**Supplementary Figure S8. Sex-biased genes and the sexual intrinsic impact on differentially expressed genes (DEGs) between APP/PS1 and WT mice**

(**A**) Principal component analysis (PCA) of samples from female and male mice (APP/PS1 and WT).

(**B**) The number of highly expressed genes in female (female-biased genes) and male (male-biased genes) mice for each cell type. We followed Jason et al. [5] to define sex-biased genes. Epi, epithelial cells; Immu, immune cells; Endo, endothelial cells; Peri. Pericytes; Fib, fibroblast; EPC, ependymal cells. Highly expressed genes in female or male animals were defined under the threshold of |log_2_fold change| > 0.1 and *p*-adjusted < 0.05.

(**C-E**) The UpSet plots for shared genes across groups of (**C**) epithelial cells, (**D**) immune cells, and (**E**) endothelial cells.

(**F**) Heatmaps displaying the expression of DEGs between APP/PS1 and WT mice of same gender in epithelial cells (*left*), immune cells (*middle*), and endothelial cells (*right*) from choroid plexus.

**Supplementary Table 1. Information on primary and secondary antibodies used in this study.**

**Supplementary Table 2. Markers of six broad cell types in ChP of mice.** pct.1, the percentage of cells where the gene is detected in the target cluster (cell type); pct.2: the percentage of cells where the gene is detected on average in other clusters.

**Supplementary Table 3. DEGs in APP/PS1 mice compared to wild-type mice.** *p*-adjusted < 0.05 and |log_2_fold change| > 0.1 were used to indicate statistical significance. To enhance visualization of a volcano plot, a constant value (such as 1E-300 for epithelial cells and 1E-25 for immune cells) was added to the *p*-adjusted values or fold change of all genes to shift the distribution of *p*-values and improve the separation of significant and non-significant genes on the plot.

**Supplementary Table 4. Markers of immune subclusters in mouse ChP.**

**Supplementary References**

1. Schwartzentruber J, Cooper S, Liu JZ, Barrio-Hernandez I, Bello E, Kumasaka N, Young AMH, Franklin RJM, Johnson T, Estrada K et al: Genome-wide meta-analysis, fine-mapping and integrative prioritization implicate new Alzheimer’s disease risk genes. Nature Genetics 2021, 53(3):392-402.

2. Reitz C, Pericak-Vance MA, Foroud T, Mayeux R: A global view of the genetic basis of Alzheimer disease. Nature Reviews Neurology 2023, 19(5):261-277.

3. Hahn O, Foltz AG, Atkins M, Kedir B, Moran-Losada P, Guldner IH, Munson C, Kern F, Pálovics R, Lu N et al: Atlas of the aging mouse brain reveals white matter as vulnerable foci. Cell 2023, 186(19):4117-4133.e4122.

4. Bader JM, Geyer PE, Müller JB, Strauss MT, Koch M, Leypoldt F, Koertvelyessy P, Bittner D, Schipke CG, Incesoy EI et al: Proteome profiling in cerebrospinal fluid reveals novel biomarkers of Alzheimer's disease. Molecular Systems Biology 2020, 16(6): e9356.

5. Rodríguez-Montes L, Ovchinnikova S, Yuan X, Studer T, Sarropoulos I, Anders S, Kaessmann H, Cardoso-Moreira M: Sex-biased gene expression across mammalian organ development and evolution. Science 2023, 382(6670):eadf1046.
